# XIAP and cIAP1 amplifications induce Beclin 1-dependent autophagy through NFκB activation

**DOI:** 10.1093/hmg/ddv052

**Published:** 2015-02-10

**Authors:** Fang Lin, Ghita Ghislat, Shouqing Luo, Maurizio Renna, Farah Siddiqi, David C. Rubinsztein

**Affiliations:** Department of Medical Genetics, Cambridge Institute for Medical Research, University of Cambridge, Wellcome/MRC Building, Addenbrooke's Hospital, Hills Road, Cambridge CB2 0XY, UK

## Abstract

Perturbations in autophagy and apoptosis are associated with cancer development. XIAP and cIAP1 are two members of the inhibitors of apoptosis protein family whose expression is elevated in different cancers. Here we report that XIAP and cIAP1 induce autophagy by upregulating the transcription of Beclin 1, an essential autophagy gene. The E3 ubiquitin ligase activity of both proteins activates NFκB signalling, leading to the direct binding of p65 to the promoter of Beclin 1 and to its transcriptional activation. This mechanism may be relevant in cancer cells, since we found increased levels of autophagy in different B-cell lymphoma-derived cell lines where XIAP is overexpressed and pharmacological inhibition of XIAP in these cell lines reduced autophagosome biogenesis. Thus, the chemotherapy resistance associated with XIAP and cIAP1 overexpression observed in several human cancers may be, at least in part, due to the Beclin 1-dependent autophagy activation by IAPs described in this study. In this context, the disruption of this increased autophagy might represent a valuable pharmacological tool to be included in combined anti-neoplastic therapies.

## Introduction

The maintenance of cancer cell survival has been associated not only with the inhibition of apoptosis, but also with the activation of macroautophagy (1). Macroautophagy, herein referred to as autophagy, is responsible for the removal of aberrant cytosolic contents by double-membraned vesicles, called autophagosomes, which deliver the sequestered material to lysosomes for degradation. In this way, the cells remain protected from the accumulation of intracellular components that may compromise cell viability (2). In the cancer context, autophagy may play opposing roles, depending on various factors such as the stage of tumour development and the set of gene mutations (that may include autophagy regulators) associated with the cancer type (3). In early stages of tumourigenesis, autophagy can play an antitumour role, and loss of positive regulators of autophagy, such as Beclin 1, Bax interacting factor-1, ultraviolet radiation resistance-associated gene, death-related kinase 1, phosphatase and tensin homolog, liver kinase B1 and Atg4c trigger tumour development (4). Indeed, even monoallelic deletion of Beclin 1, a key autophagy effector that is the orthologue of yeast *Atg6*, is sufficient to predispose to tumourigenesis (5,6). Furthermore, some negative regulators of autophagy, such as class I phosphatidylinositol 3-kinase, Akt1 and antiapoptotic members of the Bcl-2 family are oncoproteins (4). On the other hand, autophagy protects poorly vascularized tumours from cell death caused by stress conditions, such as starvation and hypoxia (7). Therefore, unlike apoptosis, which exerts a unilateral inhibitory effect on cancer development, the role of autophagy in cancer appears to be dependent on several factors, including the stage of the tumour, the mutations or the loss of genes associated with the malignancy and on the cell or tissue context. Furthermore, it is possible that the expression of certain proteins associated with cancer risk may affect both autophagy and apoptosis and the products of this interplay may be relevant to the disease.

Inhibitors of apoptosis proteins (IAPs) are important deregulators of apoptosis. They prevent cell death mainly by inactivating caspases and they also contribute to cell proliferation by modulating the activity of the Nuclear Factor κ B (NFκB) (8). X-linked inhibitor of apoptosis (XIAP) and cellular inhibitor of apoptosis 1 (cIAP1), two of the most important IAPs, are characterized by the presence of a RING finger that provides E3 ubiquitin ligase activity (9), by which they control ubiquitin signalling events, leading to the activation of NFκB, which, in turn, induces the expression of genes important for cell survival and proliferation (10).

IAPs, including XIAP and cIAP1, are overexpressed in several human cancers due to genetic alterations, abnormal activity of transcription factors controlling IAP expression and/or the absence of endogenous IAP antagonists, which contribute to the insensitivity of tumour cells towards various pharmacological treatments and unfavourable prognosis (11). For instance, high expression levels of IAPs have been associated with poor clinical outcomes of various cancers, including cervical cancers, neuroblastoma, breast cancers, melanoma, clear-cell renal carcinoma and colorectal cancer (12–18). Moreover, in haematological malignancies, high XIAP and cIAP1 levels correlate with poor prognosis of acute myelogenous leukaemia, chronic lymphocytic leukaemia and Hodgkin lymphoma (19). XIAP and cIAP1 are highly expressed in almost all of a series of 60 human cancer cell lines studied (20).

Here we describe that high levels of XIAP and cIAP1 expression induce the formation of autophagosomes by up-regulating Beclin 1 expression via the activation of the NFκB pathway. This process appears to be physiologically related to cancer state, since we found elevated levels of autophagy in various human B-cell lymphoma-derived cell lines where XIAP is overexpressed, compared with wild-type B cells. Since autophagy promotes cancer cell survival at late stages of the disease, the Beclin 1-dependent autophagy activation may contribute to the chemotherapy resistance associated with XIAP and cIAP1 overexpression found in several types of human cancer. Moreover, we showed that pharmacological inhibition of XIAP in these cell lines reduced autophagic activity and decreased their viability. Thus, disruption of this increased autophagy may be relevant for antitumour therapy.

## Results

### XIAP overexpression induces autophagy through its E3 ubiquitin ligase activity

**Figure 1.**
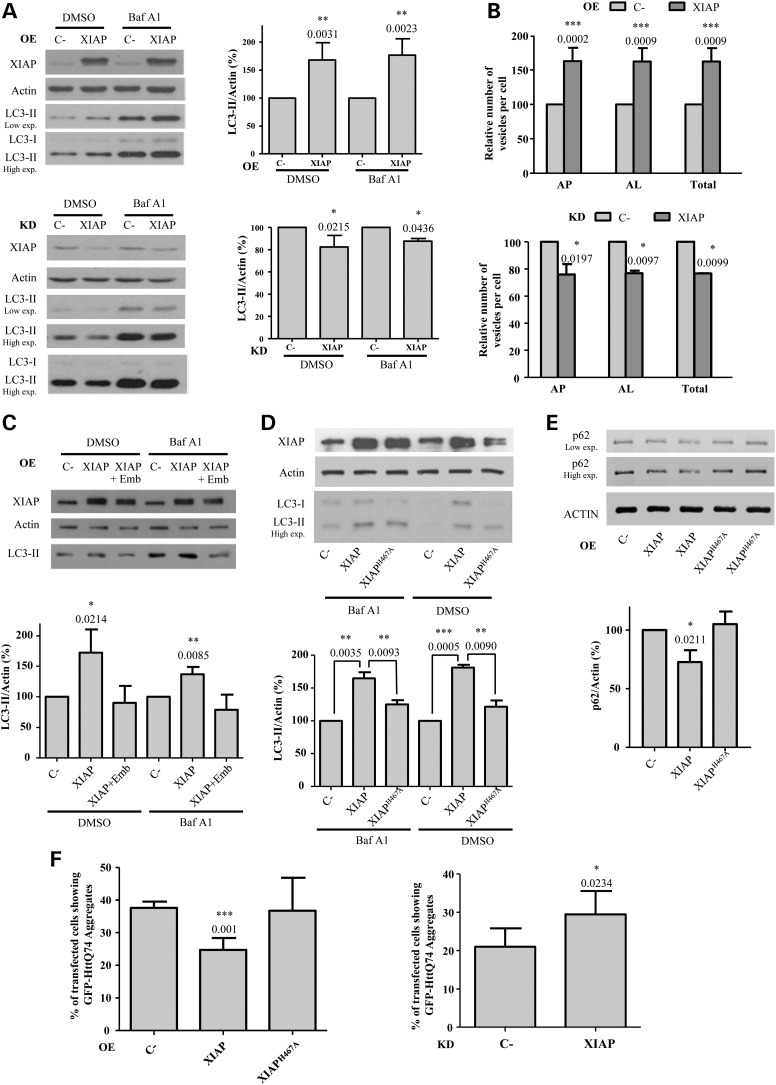
XIAP induces autophagy. (**A**) HeLa cells transfected with empty vector (C-) or XIAP expression constructs for 48 h (top panel), or with a control (C-) or XIAP siRNA for 72 h (bottom panel), were treated with DMSO or 400 nm bafilomycin A1 (Baf A1) during the last 4 h. The western blots in both panels are representative of the efficiency of XIAP overexpression (OE) and knockdown (KD) and of the levels of LC3-II in these conditions. (**B**) HeLa cells stably expressing mRFP-GFP-LC3 transfected with empty vector (C-) or XIAP expression constructs for 48 h (left panel) or with a control (C-) or XIAP siRNA for 72 h (right panel) were fixed and subjected to automatic counting of LC3 vesicles. The histograms in both panels show the percentage relative to C- of the number/cell of autophagosomes (mRFP+/GFP+) (AP), autolysosomes (mRFP+/GFP−) (AL) and both of them (total) (see also Supplementary Material, Fig. S2). (**C**) HeLa cells previously transfected with empty vector (C-) or XIAP expression constructs for 48 h were treated without or with (for cells transfected with XIAP construct) 20 μM embelin (Emb) during the last 16 h. DMSO or 400 nm bafilomycin A1 (Baf A1) were added during the last 4 h. The western blots in both panels are representative of the efficiency of XIAP overexpression (OE) and of the levels of LC3-II in these conditions. (**D**) HeLa cells previously transfected with empty vector (C-), wild-type XIAP or XIAP^H467A^ expression constructs for 48 h were treated with DMSO or 400 nm bafilomycin A1 (Baf A1) during the last 4 h. (**E**) HeLa cells previously transfected with empty vector (C-), wild-type XIAP or XIAP^H467A^ expression constructs for 48 h were subjected to western blotting. (**F**) HeLa cells were co-transfected with the GFP-HttQ74 expression construct plus empty vector (C-), wild-type XIAP or XIAP^H467A^ expression constructs for 48 h (left panel) or with a control (C-) or XIAP siRNA, where 24 h later the cells were transfected with the GFP-HttQ74 expression construct for 48 h (right panel). In both panels, the cells were then fixed and the percentage of transfected cells with aggregates was calculated as shown in the histograms. At least 150 cells were counted per sample (see also Supplementary Material, Fig. S4). Densitometric measurements of LC3-II or p62 bands were normalized to the corresponding actin bands in the corresponding histograms. The values shown in all the histograms represent the mean ± standard deviation from at least three independent experiments performed in triplicate samples/condition. The *P*-values were determined using Student's *t*-test. See also Supplementary Material, Figure S1.

Since XIAP amplifications are associated with cancers, we first examined the effect of the overexpression of this protein on the levels of the microtubule associated protein 1 light chain 3 (LC3-II), a well-established marker of autophagy (21). The levels of LC3-II are indicative of the number of autophagosomes. In the presence of potent inhibitors of lysosomal degradation, such as bafilomycin A1 (Baf A1), LC3-II is not degraded and thus its changes resulting from other perturbations can be attributed to alterations in LC3-II synthesis (22,23). We found that the overexpression of XIAP in HeLa cells caused a substantial increase in LC3-II levels in both the absence and presence of Baf A1 (Fig. 1A, top), which suggests that XIAP promotes the formation of autophagosomes. In contrast, XIAP knockdown caused a slight decrease in LC3-II levels (Fig. 1A, bottom). To rule out the possibility of an off-target effect of the Smartpool siRNAs, we confirmed that LC3-II levels decreased with two different deconvoluted siRNAs targeted against XIAP (Supplementary Material, Fig. S1A). Since the Smartpool showed the greatest silencing efficiency, we used it in all the subsequent knockdown experiments. The decrease of LC3-II levels caused by XIAP knockdown was also observed in human neuroblastoma SK-N-SH cells (Supplementary Material, Fig. S1B) and in MCF10A cells, where we also confirmed that we could rescue the negative effects of XIAP knockdown by overexpressing XIAP in knockdown cells (Supplementary Material, Fig. S1C).

We further confirmed the effect of XIAP on autophagosome formation using another autophagy assay, based on the sensitivity of GFP relative to RFP to the acidic lysosomal environment. Hence, cells stably expressing a monomeric RFP (mRFP)-GFP-LC3 tandem reporter trace autophagosome maturation by discriminating autophagosomes that show both red and green fluorescence, compared with autolysosomes that display only red signals (24). Consistent with our previous results (Fig. 1A), XIAP overexpression increased (≈ 62% more) both autophagosomes and autolysosomes (Fig. 1B, top) (see also Supplementary Material, Fig. S2), whereas XIAP knockdown caused the opposite effect to a lesser extent (≈ 25% less) (Fig. 1B, bottom).

In order to further confirm the significance of the high levels of XIAP on autophagy activation, we used embelin, a specific inhibitor of XIAP that prevents its proliferative and antiapoptotic activities (25). Embelin concentrations ranging from 10 to 50 µm are required for effective inhibition of NFκB signalling pathway (26) in various cancer cell lines. This inhibitor, at a concentration range of 10–20 µm, mildly impaired autophagy in HeLa cells (Supplementary Material, Fig. S3A) and in mouse embryonic fibroblasts (Supplementary Material, Fig. S3B). However, the increase in the level of LC3-II caused by XIAP overexpression was completely abolished by 20 µm embelin (Fig. 1C), supporting the importance of amplified XIAP activity for autophagy activation. Embelin concentrations of 200 nm and below [which would not be predicted to impact on NFκB signalling] (26) did not affect LC3-II levels in HeLa (Supplementary Material, Fig. S3C) and mouse embryonic fibroblasts (MEFs) (Supplementary Material, Fig. S3D). In MCF10A cells, the inhibitory effect of embelin on autophagy is concentration-dependent. While it actually increased LC3-II levels at 10 µm (Supplementary Material, Fig. S3E), this was associated with decreased MCF10A cell viability (Supplementary Material, Fig. S3F). However, at 5 µm where cell viability is not affected (Supplementary Material, Fig. S3F), it decreased the levels of LC3-II (Supplementary Material, Fig. S3G), consistent with our knockdown data in MCF10A cells and other lines (Fig. 1A, Supplementary Material, Fig. S1A–C), suggesting that the effects of 10 µm embelin in these cells was off-target.The activities of XIAP and cIAP1 family members rely on the presence of a RING finger domain that provides E3 ubiquitin ligase activity (9), by which they can modulate the expression of genes important for cell survival and proliferation through the NFκB pathway (10). In order to discern whether the induction of autophagy by XIAP overexpression is dependent on its E3 ubiquitin ligase activity, we transfected the cells with a XIAP^H467A^ mutant defective in this activity (9), which increased LC3-II levels far less than the wild-type XIAP (Fig. 1D). We assessed then the impact of the overexpression of this mutant on the levels of p62 (SQSTM1/sequestosome 1), an endogenous autophagy substrate (27). The overexpression of wild-type XIAP, but not of XIAP^H467A^, decreased the levels of p62 (Fig. 1E). Mutant huntingtin (Htt) Q74 is another well-established autophagy substrate. The proportion of cells with Q74 aggregates is a direct function of levels of the protein and inversely correlates with autophagic activity (28). Consistent with our previous data, the percentage of cells with mutant htt aggregates decreased with overexpression of wild-type XIAP and not of XIAP^H467A^ (Fig. 1F, left graph) (see also Supplementary Material, Fig. S4A), whereas XIAP knockdown led to an accumulation of cells with htt aggregates (Fig. S 1F, right graph) (see also Supplementary Material, Fig. S4B).

### XIAP overexpression upregulates Beclin 1 levels through the activation of NFκB signalling

**Figure 2.**
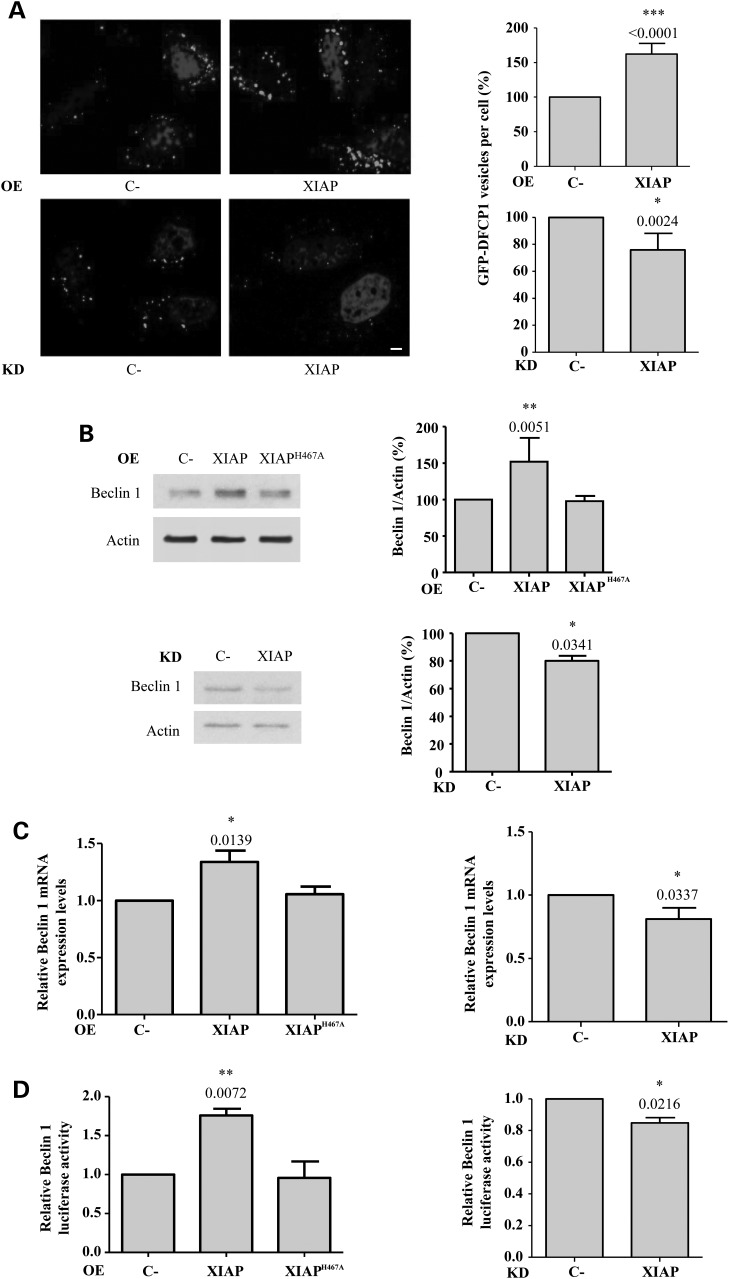
XIAP up-regulates the levels of Beclin 1. (**A**) HeLa cells stably expressing GFP-DFCP1 were transfected with empty vector (C-) or wild-type XIAP expression constructs for 48 h and were then fixed. The GFP-DFCP1 vesicles were counted using a confocal microscope. Representative images of cells displaying GFP-DFCP1 vesicles are shown. The percentage of GFP-DFCP1 vesicles per cell relative to C- cells is shown in the histograms on the left. Bar, 10 µm. (**B**) (top panel) HeLa cells were transfected with empty vector (C-), wild-type XIAP or XIAP^H467A^ expression constructs for 48 h; (bottom panel) HeLa cells were transfected with a control (C-) or XIAP siRNA for 72 h. Densitometric measurements of Beclin 1 bands were normalized to the corresponding actin bands and are shown in the histograms on the right. (**C**) (left panel) mRNA from HeLa cells previously transfected with empty vector (C-), wild-type XIAP or XIAP^H467A^ expression constructs for 48 h was analysed by qRT-PCR for Beclin 1-actin mRNA; (right panel) RNA from HeLa cells previously transfected with a control (C-) or XIAP siRNA for 72 h was analysed by qRT-PCR for Beclin 1-actin mRNA. In both panels, the levels of Beclin 1 mRNA were normalized to actin mRNA levels. (**D**) (left panel) HeLa cells were co-transfected with CHET4-luciferase reporter containing the Beclin 1 promoter plus empty vector (C-), wild-type XIAP or XIAP^H467A^ expression constructs for 48 h; (right panel) HeLa cells were transfected with a control (C-) or XIAP siRNA. Twenty-four hours later, cells were transfected with CHET4-luciferase reporter containing the Beclin 1 promoter for 48 h. In both panels, values of the relative luciferase activity are reported in the histograms. See also Supplementary Material, Figure S2.

Autophagosome precursors, called omegasomes, contain phosphatidylinositol-3-phosphate (PI(3)P) and can be identified as structures which bind the PI(3)P-binding protein DFCP1 (double FYVE domain-containing protein 1) (29). Consistent with its effect on autophagy, XIAP overexpression increased the number of GFP-DFCP1 positive dots, whereas XIAP knockdown caused the opposite effect (Fig. 2A).

The translocation of DFCP1 to early autophagic vesicles is dependent on Beclin 1 (29). The E3 ubiquitin ligase properties of XIAP activate NFκB (8), and NFκB has been reported to stimulate Beclin 1 transcription (30). Thus, we investigated whether XIAP stimulates autophagy via the NFκB-mediated up-regulation of Beclin 1 expression. Beclin 1 enhances the conjugation of Atg12 to Atg5, two autophagy-related proteins involved in the early stages of autophagosome biosynthesis (31), and the levels of this conjugate were increased after XIAP overexpression (Supplementary Material, Fig. S5A), which also up-regulated Beclin 1 protein (Fig. 2B, top) and mRNA (Fig. 2C, left) levels. XIAP knockdown reduced Beclin 1 protein (Fig. 2B, bottom) and mRNA (Fig. 2C, right) levels. XIAP^H467A^ failed to increase Beclin 1 levels (Fig. 2B, top and 2C, left). This suggests that the induction of Beclin 1 expression by XIAP is mediated by its E3 ubiquitin ligase activity. Consistent with these results, the overexpression of wild-type but not XIAP^H467A^ enhanced the transcriptional activation of a Beclin 1 promoter reporter (Fig. 2D, left), whereas XIAP knockdown slightly decreased it (Fig. 2D, right). All together, these data show that XIAP upregulates Beclin 1 transcription through its E3 ubiquitin ligase activity. p53 levels were not altered by overexpression of XIAP and XIAP^H467A^ in HeLa cells (Supplementary Material, Fig. S5B), or by XIAP overexpression in MCF10A cells (Supplementary Material, Fig. S5C).

**Figure 3.**
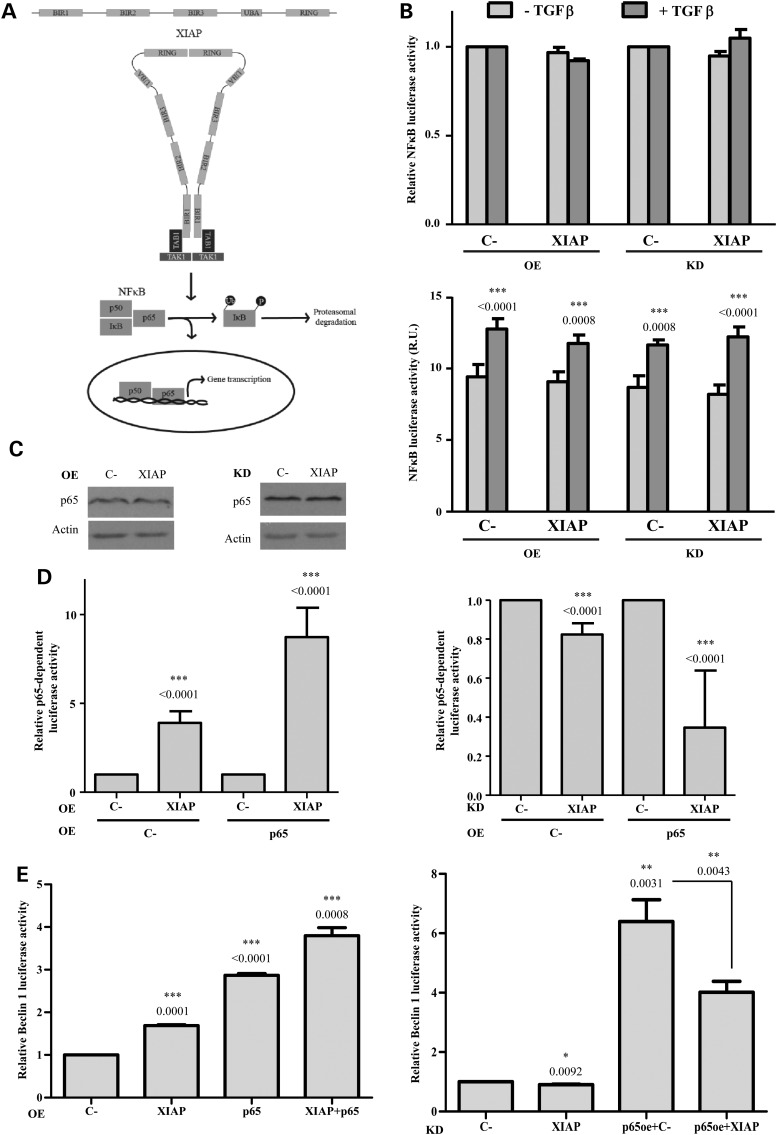
XIAP induces Beclin 1 transcription via p65/NFκB activation. (**A**) Schematic diagram of XIAP-mediated NFκB activation. BIR1 and RING domains of two XIAPs interact leading to dimerization that recruits TAB1. TAK1 is consequently activated by interaction with this complex in the form of dimers, which triggers NFκB signalling. This occurs after the phosphorylation of IκB and its resultant ubiquitination and proteasomal degradation. In this way, the p50/p65 heterodimer is translocated to the nucleus, where gene transcription occurs. (**B**) For overexpression (OE) bars, HeLa cells were co-transfected with a luciferase reporter containing the NFκB promoter plus empty vector (C-) or XIAP expression constructs for 48 h. For knockdown (KD) bars, HeLa cells were transfected with a control (C-) or XIAP siRNA. Twenty-four hours later, cells were transfected with a luciferase reporter containing the NFκB promoter for 48 h. In all cases, cells were treated or not with 2 µg/ml TGFβ for 2 h. The mean values of the relative luciferase activity are reported in the histograms. (**C**) HeLa cells were transfected with empty vector (C-) or XIAP expression constructs for 48 h (left panel) or with a control (C-) or XIAP siRNA for 72 h (right panel). The western blots in both panels are representative of at least three independent experiments performed in triplicate. (**D**) (left panel) HeLa cells were co-transfected for 48 h with a luciferase reporter that contains a promoter with p65 binding site plus empty vector (C-) or XIAP expression constructs. In both cases (C- and XIAP), an empty vector or a p65 expression constructs was included in the co-transfection; (right panel) HeLa cells were transfected for 48 h with a control (C-) or XIAP siRNA. Twenty-four hours later, cells were co-transfected with a luciferase reporter that contains a promoter with p65 binding site plus empty vector (C-) or p65 expression constructs. In both panels, values of the relative luciferase activity are reported in the histograms. (**E**) (left panel) HeLa cells were co-transfected for 48 h with CHET4-luciferase reporter containing the Beclin 1 promoter plus empty vector (C-) or XIAP expression constructs. In both cases (C- and XIAP), an empty vector or a p65 expression constructs was included in the co-transfection; (right panel) HeLa cells were transfected for 48 h with a control (C-) or XIAP siRNA. Twenty-four hours later, cells were co-transfected with CHET4-luciferase reporter containing the Beclin 1 promoter plus empty vector (C-) or p65 expression constructs. In both panels, values of the relative luciferase activity are reported in the histograms. The values shown in all the histograms represent the mean ± standard deviation from at least three independent experiments performed in triplicate samples/condition. The *P*-values were determined using Student's *t*-test. See also Supplementary Material, Figure S3.

Although the detailed mechanism by which XIAP mediates NFκB activation is not completely understood, it is now well-established that XIAPs can form dimers through interactions between their RING and BIR1 domains, which lead to the binding of the transforming growth factor-beta (TGFβ) activated kinase 1 (TAK1) adaptor protein, TAB1 (as schematically detailed in Fig. 3A). TAK1 is then recruited to this complex and facilitates its dimerization and consequent activation, which triggers the NFκB signalling pathway (32). In both steady state and stimulated conditions with TGFβ that activates NFκB through TAK1 (33), neither the overexpression of XIAP nor its knockdown affected the transcriptional activation of the NFκB promoter (Fig. 3B, top). The effect of TGFβ on the transcriptional activation of the NFκB promoter is shown in the bottom graph, where the values are not normalized to the control (C-) samples (Fig. 3B, bottom). The protein levels of p65, a transcriptional activator in the NFκB complex, did not change after the overexpression or knockdown of XIAP (Fig. 3C). However, the expression of an NFκB-dependent promoter reporter was increased after XIAP overexpression (Fig. 3D, left) and slightly, but significantly reduced upon XIAP knockdown (Fig. 3D, right). Moreover, the overexpression and knockdown of XIAP, respectively, enhanced and reversed the transactivation of this NFκB-dependent promoter reporter by overexpression of p65 (Fig. 3D). This transcription factor has been reported to up-regulate Beclin 1 transcription (30). We thus investigated if p65 was involved in the XIAP-mediated transcriptional activation of Beclin 1 that we had observed (Fig. 2D). Indeed, overexpression of XIAP enhanced the amplification of Beclin 1 transcriptional activation by p65 (Fig. 3E, left), while XIAP knockdown reversed the effect (Fig. 3E, right).

**Figure 4.**
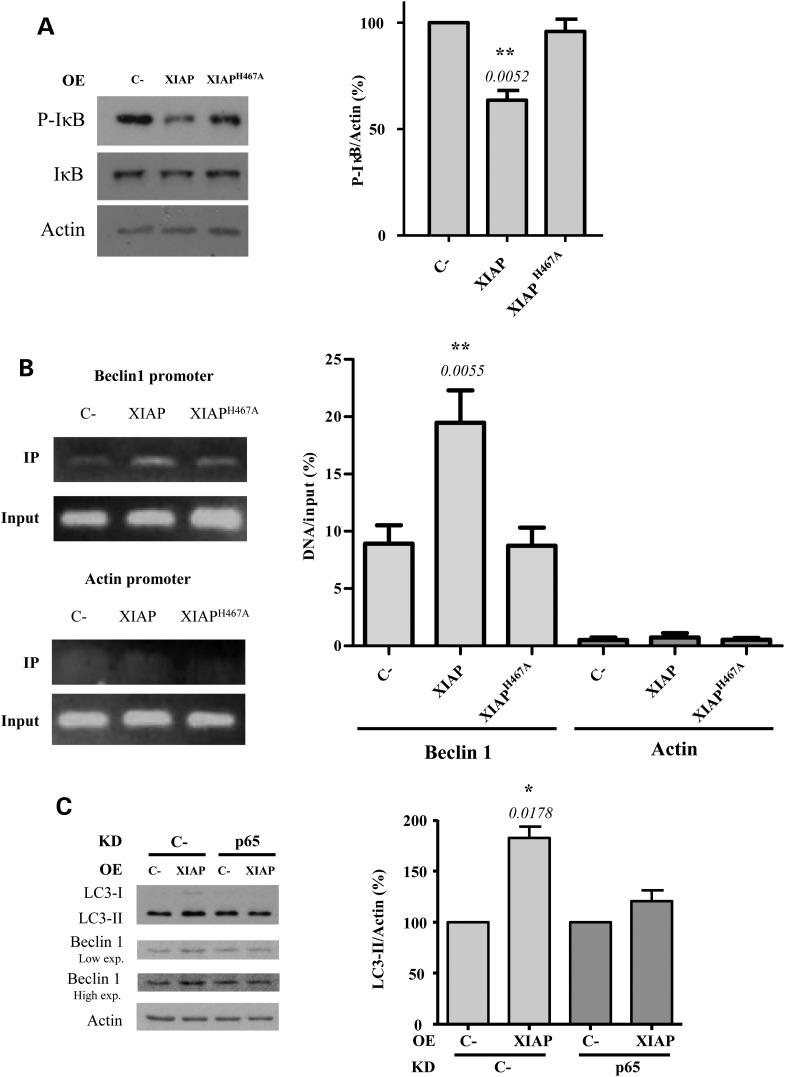
p65 is involved in the activation of autophagy by XIAP. (**A**) HeLa cells previously transfected with empty vector (C-), wild-type XIAP or XIAP^H467A^ expression constructs for 48 h were subjected to western blotting to detect P-IκB and IκB levels. The blots are from the same set of experiments. Densitometric measurements of phospho-IκB (P-IκB) bands were normalized to the corresponding bands of actin and are shown in the histogram on the right. (**B**) HeLa cells previously transfected with empty vector (C-), wild-type XIAP or XIAP^H467A^ expression constructs for 48 h were subjected to a ChiP assay. The amount of *in vivo* binding of endogenous p65 to Beclin 1 and actin (as a negative control) promoters was quantified by real-time PCR. Data are representative of three independent experiments. (**C**) HeLa cells were transfected for 48 h with a control (C-) or p65 siRNA. Twenty-four hours later, cells were co-transfected for 48 h with an empty vector (C-) or XIAP expression constructs. Cells were treated with DMSO or 400 nm bafilomycin A1 during the last 4 h. Densitometric measurements of LC3-II bands were normalized to the corresponding actin bands and are shown in the histograms on the right. The values shown in all the histograms represent the mean ± standard deviation from at least three independent experiments performed in triplicate samples/condition. The *P*-values were determined using Student's *t*-test. See also Supplementary Material, Figure S4.

The transcriptional activity of p65 is triggered once the inhibitor of NFκB proteins (IκB) is phosphorylated, which leads to its dissociation from the NFκB complex. This phosphorylation enables ubiquitination of IκB which enables its degradation by the proteasome (34) (Fig. 3A). We found that the levels of the phosphorylated form of IκB decreased after overexpression of wild-type XIAP, but not XIAP^H467A^ (Fig. 4A). This decrease was reversed in the presence of the proteasome inhibitor MG132 (Supplementary Material, Fig. S6A), suggesting that XIAP overexpression induces the proteasomal degradation of IκB, mainly through its E3 ubiquitin ligase activity. IκB prevents the translocation of the NFκB dimer p50/p65 from the cytosol to the nucleus where it binds relevant promoters (34). Consistent with this model, overexpression of wild-type XIAP increased the amount of p65 binding to the endogenous Beclin 1 promoter (assessed by chromatin immunoprecipitation), whereas the overexpression of XIAP^H467A^ failed to reproduce this effect (Fig. 4B). Indeed, p65 knockdown (we verified the p65 knockdown by immunocytochemistry as shown in Supplementary Material, Fig. S6B), attenuated the positive effect of XIAP overexpression on both Beclin 1 and LC3-II levels (Fig. 4C), confirming the importance of p65 in the XIAP-mediated activation of Beclin 1-dependent autophagy.

### XIAP amplification in some large B-cell lymphoma cell lines is associated with increased autophagy

**Figure 5.**
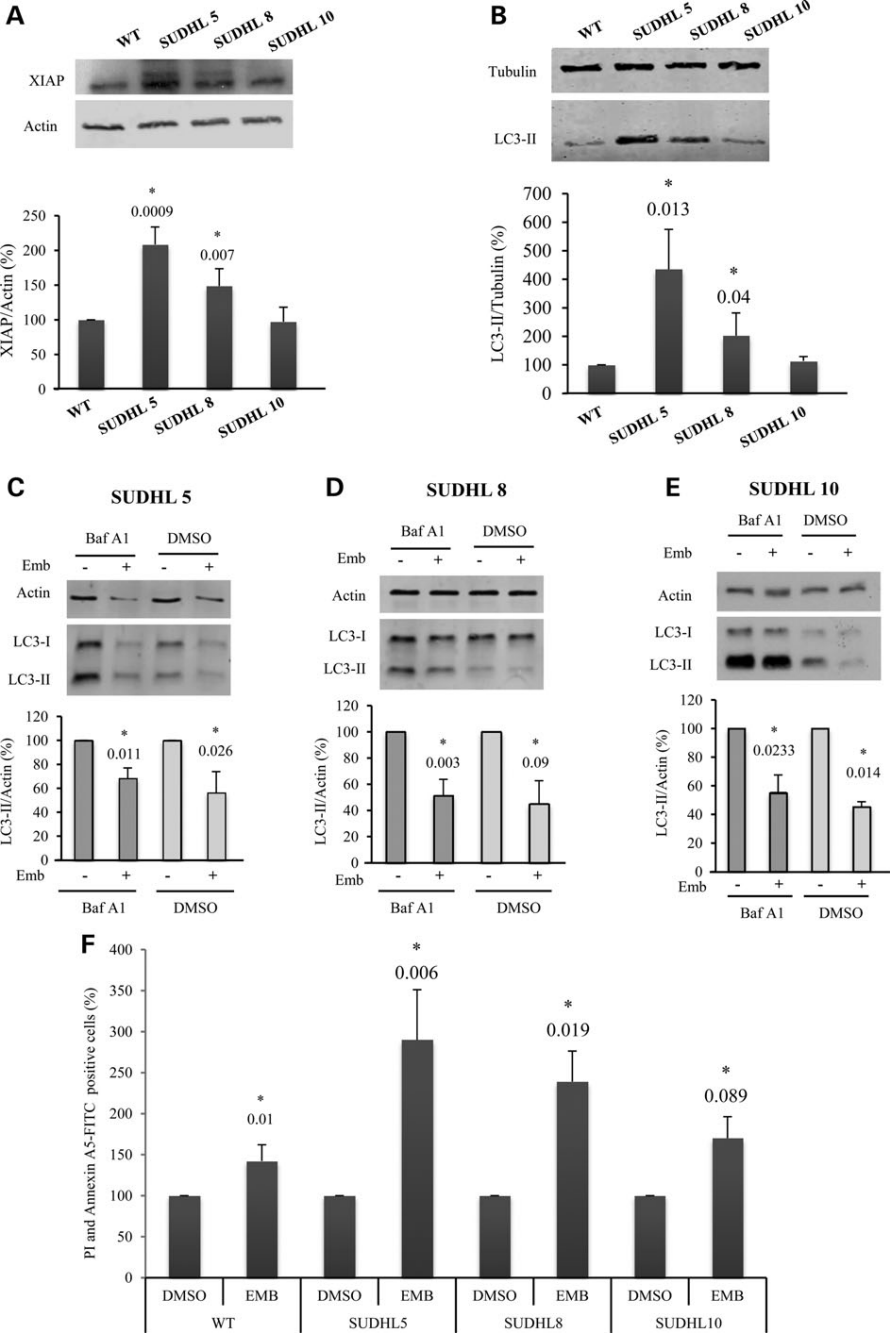
High levels of XIAP in B-cell lymphoma activate autophagy. (**A**) B cells (WT) and three diffuse large B-cell lymphoma cell lines, SUDHL5, SUDHL8 and SUDHL10 were subjected to immunoblotting with XIAP and actin antibodies. (**B**) The same cell lines were treated with 400 nm bafilomycin A1 and were subsequently subjected to immunoblotting with LC3 and tubulin antibodies. The diffuse large B-cell lymphoma cell lines, SUDHL5 (**C**), SUDHL8 (**D**) and SUDHL10 (**E**), were treated without or with 10 μM embelin (Emb) for 16 h, and treated without or with 400 nm bafilomycin A1 (Baf A1) for the last 4 h of the experiment, and were subsequently subjected to immunoblotting with LC3 and tubulin antibodies. (**F**) Propidium iodide and FITC-conjugated Annexin A5 staining detected by flow cytometry of B cells (WT) SUDHL5, SUDHL8 and SUDHL10 (5, 8 and 10) treated without or with 10 μM embelin (Emb) for 16 h. The percentage of cells positive for both PI and Annexin A5 are shown. Densitometric measurements of LC3-II bands were normalized to the corresponding actin or tubulin bands and are shown in the corresponding histograms. The values shown in all the histograms represent the mean ± standard deviation from at least three independent experiments performed in triplicate samples/condition. The *P*-values were determined using one sample *t*-tests, where controls are set to 100%. See also Supplementary Material, Figure S7.

To discern whether the XIAP effect on autophagy is relevant in a cancer context, we assessed LC3-II levels in diffuse large B-cell lymphoma cell lines, where XIAP was reported to be overexpressed and associated with poor clinical outcomes (35). We found that XIAP levels were significantly higher in two of these cell lines (SUDHL5 and SUDHL8), compared with wild-type B cells, while the XIAP levels of the third line (SUDHL10), were hardly elevated (Fig. 5A). The levels of XIAP in these cell lines correlated with autophagosome formation as assessed by LC3-II levels in the presence of Baf A1 (Fig. 5B). As predicted, embelin decreased LC3-II levels in all of these patient cell lines (Fig. 5C–E). Furthermore, embelin appeared to increase apoptosis in these cell lines in a manner that appeared to correlate with both XIAP levels and autophagic activity (Fig. 5F and Supplementary Material, Fig. S7). This result is compatible with a role for XIAP-autophagy in the viability of these lymphoma cell lines.

### cIAP1 overexpression induces Beclin 1-dependent autophagy through the activation of NFκB signalling

**Figure 6.**
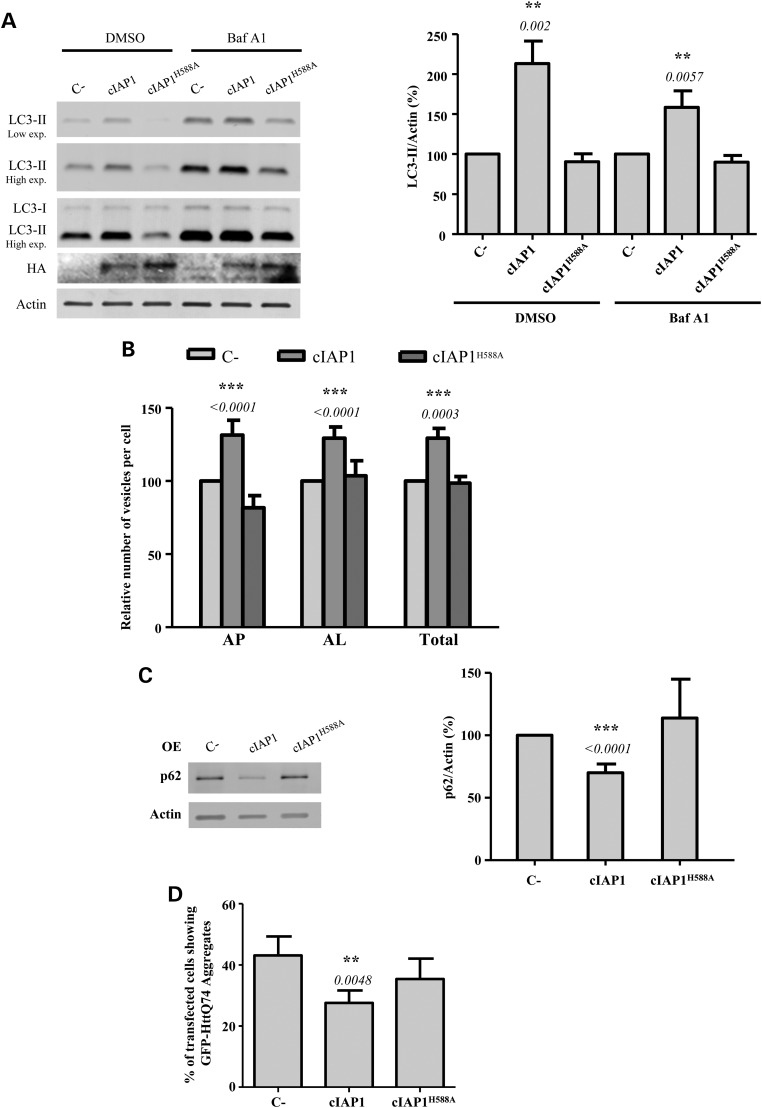
cIAP1 overexpression induces autophagy. (**A**) HeLa cells previously transfected with empty vector (C-), wild-type cIAP1 or cIAP1^H588A^ expression constructs for 48 h were treated with DMSO or 400 nm bafilomycin A1 (Baf A1) during the last 4 h. Blots were probed with the indicated antibodies and the HA indicates the cIAP1 constructs. Densitometric measurements of LC3-II bands were normalized to the corresponding actin bands and are shown in the histogram on the right. (**B**) HeLa cells stably expressing mRFP-GFP-LC3 transfected with empty vector (C-), wild-type cIAP1 or cIAP1^H588A^ expression constructs for 48 h were fixed and subjected to automatic counting of LC3 vesicles. The histogram shows the percentage relative to C- of the number/cell of autophagosomes (mRFP+/GFP+) (AP), autolysosomes (mRFP+/GFP-) (AL) and both of them (total). (**C**) HeLa cells previously transfected with empty vector (C-), wild-type cIAP1 or cIAP1^H588A^ expression constructs for 48 h were subjected to western blotting. Densitometric measurements of p62 bands were normalized to the corresponding actin bands and are shown in the histogram on the right. (**D**) HeLa cells were co-transfected with the GFP-HttQ74 expression construct plus empty vector (C-), wild-type cIAP1 or cIAP1^H588A^ expression constructs for 48 h. The cells were then fixed and the percentage of transfected cells with aggregates was calculated as shown in the histogram. At least 150 cells were counted per sample. The values shown in all the histograms represent the mean ± standard deviation from at least three independent experiments performed in triplicate samples/condition. The *P*-values were determined using Student's *t*-test. See also Supplementary Material, Figure S8.

cIAP1 is another important member of the IAP family. This protein also harbours a RING finger domain by which it regulates the ubiquitin-dependent activation of NFκB signalling pathway (36). We investigated the effect of this protein on autophagy by overexpressing wild-type cIAP1 or the cIAP1^H588A^ mutant, defective in its E3 ubiquitin ligase activity. Figure 6A shows that the levels of LC3-II increased in HeLa cells after overexpression of wild-type cIAP1, but not of cIAP1^H588A^. This result was also observed in HCT-116 cells (Supplementary Material, Fig. S8A) and in MEFs (Supplementary Material, Fig. S8B). Moreover, the number of autophagosomes and autolysosomes in cells stably expressing GFP-mRFP-LC3 increased upon the overexpression of cIAP1, but not of cIAP1^H588A^, which indicates that this protein induces autophagy through its E3 ubiquitin ligase activity (Fig. 6B; see also Supplementary Material, Fig. S9A). cIAP1 overexpression also decreased p62 levels in both HeLa (Supplementary Material, Fig. S6C) and HCT-116 (Supplementary Material, Fig. S8C) cells, an effect not observed upon cIAP1^H588A^ overexpression. Furthermore, the percentage of cells with mutant htt aggregates decreased after overexpression of wild-type cIAP1 but not of cIAP1^H588A^ (Fig. 6D, see also Supplementary Material, Fig. S9B). As we observed with XIAP, collectively these data support the relevance of the E3 ubiquitin ligase activity of cIAP1 in autophagy activation.

**Figure 7.**
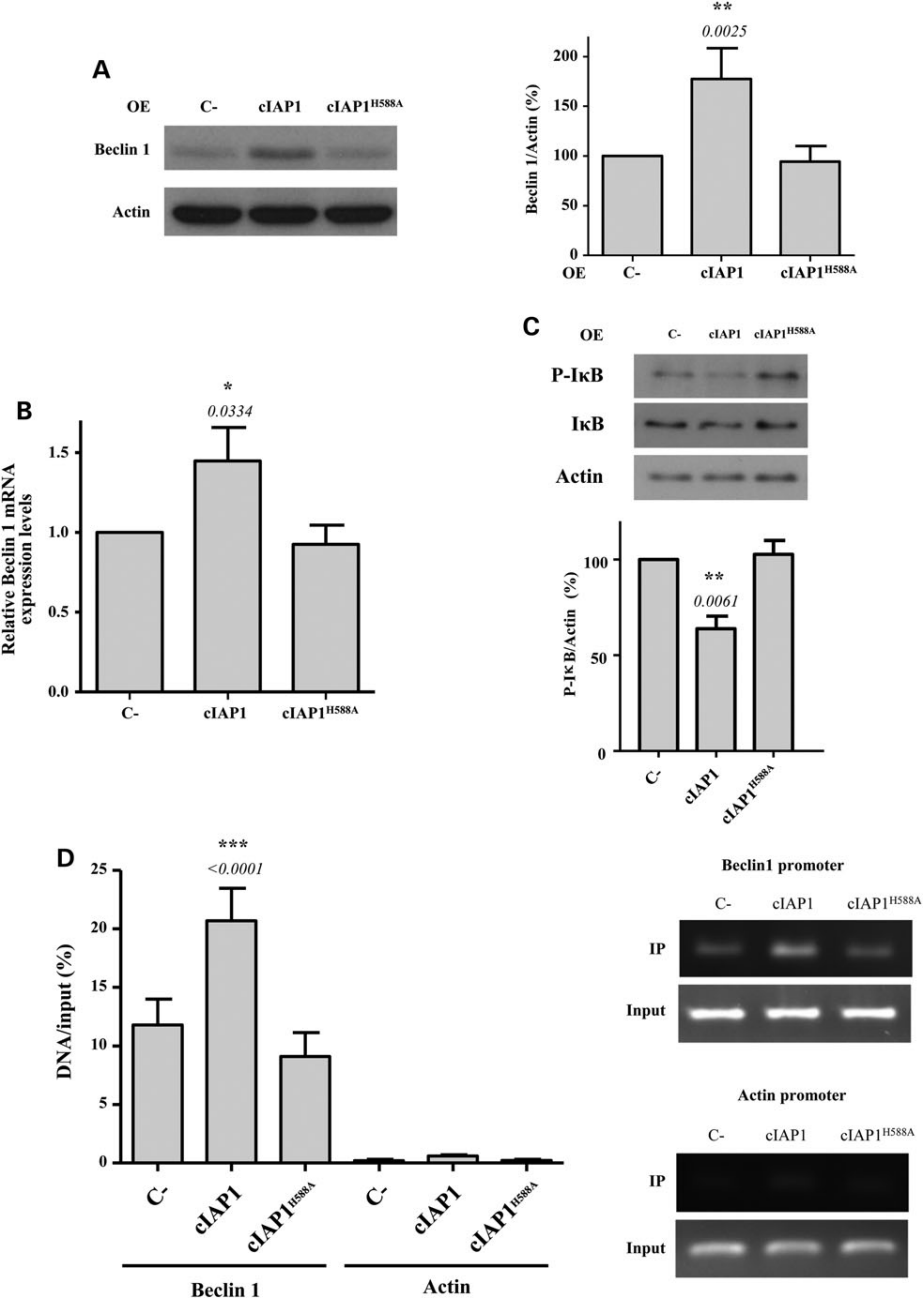
cIAP1 induces Beclin 1 transcription via p65/NFκB activation. (**A**) HeLa cells were transfected with empty vector (C-), wild-type cIAP1 or cIAP1^H588A^ expression constructs for 48 h. Densitometric measurements of Beclin 1 bands were normalized to the corresponding actin bands and are shown in the histograms on the right. (**B**) mRNA from HeLa cells previously transfected with empty vector (C-), wild-type cIAP1 or cIAP1^H588A^ expression constructs for 48 h was analysed by qRT-PCR for Beclin 1-actin mRNA. The levels of Beclin 1 mRNA were normalized to Actin mRNA levels. (**C**) HeLa cells previously transfected with empty vector (C-), wild-type cIAP1 or cIAP1^H588A^ expression constructs for 48 h were subjected to western blotting to detect P-IκB and IκB levels. The blots shown are from the same set of experiments. Densitometric measurements of phospho-IκB (P-IκB) bands were normalized to the corresponding bands of actin and are shown in the histogram below. (**D**) HeLa cells previously transfected with empty vector (C-), wild-type cIAP1 or cIAP1^H588A^ expression constructs for 48 h were subjected to a ChiP assay. The amount of *in vivo* binding of endogenous p65 to Beclin 1 and actin (as a negative control) promoters was quantified by real-time PCR. Data are representative of three independent experiments. The values shown in all the histograms represent the mean ± standard deviation from at least three independent experiments performed in triplicate samples/condition. The *P*-values were determined using Student's *t*-test.

Given that cIAP1 was also reported to regulate the NFκB signalling pathway through its E3 ubiquitin ligase activity (36), we further investigated and confirmed that the expression levels of Beclin 1 increased when wild-type cIAP1 but not cIAP1^H588A^ was overexpressed (Fig. 7A). The mRNA levels of Beclin 1 also increased upon the overexpression of wild-type cIAP1 only (Fig. 7B), which confirms that the E3 ubiquitin ligase activity of cIAP1 is important for the up-regulation of Beclin 1 transcription. The overexpression of cIAP1 resulted in a decrease of the phosphorylated form of IκB (Fig. 7C), which is the form of IκB that is usually degraded by the proteasome (34). The proteasome inhibitor, MG132, reversed the decrease of phospho-IκB levels caused by the overexpression of the wild-type cIAP1 (Supplementary Material, Fig. S6). Conversely, cIAP1^H588A^ overexpression failed to decrease phospho-IκB. Finally, chromatin immunoprecipitation (ChIP) analysis also confirmed that wild-type cIAP1 increased p65 binding to the Beclin 1 promoter, an effect not seen with cIAP1^H588A^ (Fig. 7D).

## Discussion

XIAP and cIAP1 are amplified in various cancers and here we have shown that overexpression of these proteins induces autophagy. One of the most important contributions of IAPs to cell survival and tumourigenesis resides in their ability to activate the NFκB signalling pathway (36), which also drives the effects of these IAPs on autophagy via their E3 ubiquitin ligase activities. Consistent with these data, NFκB activation by XIAP and cIAP1 requires a ubiquitin-dependent signalling pathway and the RING domain of both proteins that harbours their E3 ubiquitin ligase activity (36). NFκB, in turn, activates autophagy by up-regulating the transcription of Beclin 1, a key autophagy gene (37). Hence, our data link NFκB signalling and Beclin 1-dependent autophagy under the control of the E3 ubiquitin ligase activity of XIAP and cIAP1. Since Beclin 1 is an important autophagy gene, analysing the effect of XIAP perturbations on autophagy in cells with Beclin 1 knockdown was not possible. Therefore, we cannot discard the possibility of additional mechanisms independent of Beclin-1 for autophagy activation by XIAP. The effects of XIAP knockdown on autophagy are modest, compared with the overexpression effects, suggesting that the major relevance of this gene in autophagy is when it is amplified in cancers. Indeed, we demonstrated this in lymphoma cell lines.

In contrast to what we have described, a previous study reported that XIAP inhibited autophagy by upregulating p53 levels via the inhibition of its degradation by Mdm2 (38). The results of this report were based on the knockdown of XIAP and on the use of embelin. However, they used very low concentrations of embelin (50–200 nm), far below its effective concentration [10–40 µm for apoptosis activation (25) and 10–50 µm are required for effective inhibition of NFκB signalling pathway (26)]. They used MEFs and MCF10A cells for their studies and obtained different results to ourselves, where we also used these cells. However, their study focussed on the effects of XIAP knockdown, while we have stressed the consequences of amplification in the cancer context, where we showed that autophagy is inhibited by embelin in cancer cells lines with XIAP amplification. We did not observe any effect of XIAP overexpression on p53 levels, suggesting that the mechanism proposed for the knockdown effects were not contributing to the overexpression context. We also observed similar phenomena with cIAP1 overexpression as we did with XIAP, with consistent mechanistic overlaps.

The higher expression levels of XIAP and cIAP1 in some cancer cells may contribute to tumour maintenance not only via the inhibition of apoptosis, but also through the activation of autophagy. Likewise, in relevant human cancers, the inhibition of autophagy may be a useful tool to eliminate the chemotherapy resistance due to apoptosis inhibition by XIAP and cIAP1 overexpression. Consistent with this concept, the inactivation of IAPs, especially when combined with other treatments, may result in preferential death of tumour cells, compared with normal cells (36,39,40).

The roles of autophagy in cancer appear to be context-dependent (41). Beclin 1 is functionally a haploinsufficient tumour suppressor gene in mice and is monoallelically deleted in some sporadic breast, ovarian and prostate cancers (5,6), although its role as a haploinsufficient tumour suppressor in cancer patients has been questioned as these deletions appear to also invariably include loss of *BRCA1* (42). Furthermore, there appear to be complexities as to whether p53 activity impacts or not on the therapeutic effects of autophagy inhibition in pancreatic cancer models (3,43). Therefore, we appreciate that the definitive causal contributions of the XIAP/cIAP1-Beclin 1-autophagy pathway to cancer still will require further studies.

All together, these observations further highlight the critical role played by the high levels of XIAP in cancerous cells. Our findings suggest that overexpression of IAPs in cancers has biological relevance in controlling not only apoptosis (36), but also the autophagic response, which may both impact on therapy.

## Materials and Methods

### Cell culture

HeLa cells, MEFs and SKNSH were cultured at 37°C, 5% CO_2_ in 10% FBS, 2 mm l-glutamine and 100 U/ml penicillin/streptomycin supplemented Dulbecco's modified Eagle's medium (DMEM) D6546 (Invitrogen). HeLa cells stably expressing mRFP-GFP-LC3 were maintained in similar media supplemented with 600 mg/ml of G418. MCF10A were from Horizon Discovery and were grown at 37°C, 5% CO_2_ in DMEM including 2.5 mm l-glutamine and 15 mm HEPES, supplemented with 5% horse serum, 10 µg/ml insulin, 20 ng/ml hEGF, 0.5 µg/ml hydrocortisone and 0.1 µg/ml cholera toxin. Wild-type B-cells and human diffuse large B-cell lymphoma cell lines (DLBCL) SUDHL5, SUDHL8 and SUDHL10 [obtained from Deutsche Sammlung von Mikroorganismen und Zellkulturen, Braunschweig, Germany (DSMZ)] were cultured at 37°C, 5% CO_2_ in 10% FBS, 2 mm l-glutamine and 100 U/ml penicillin/streptomycin supplemented RPMI 1640 (Invitrogen) (see Supplementary Material online).

### DNA constructs

pcDNA3.1-XIAP-Myc was provided by G.S. Salvesen (Addgene plasmid 11833), pEBB-XIAP^H588A^ was provided by J.D. Ashwell (Addgene plasmid 11559) (9), pEBB-HA-cIAP1 and pEBB-HA-cIAP1^H588A^ were provided by C.S. Duckett (Addgene plasmids 38232 and 38233, respectively) (44). pcDNA3.1 and pEBB empty vectors were used as controls. The first exon of the huntingtin protein with 74 polyglutamines, tagged with EGFP, EGFP-HttQ74 has been extensively characterized (28). The CHET4-luciferase reporter containing the Beclin 1 promoter (see Supplementary Material, Fig. S9A) was provided by C. Schneider (30). The luciferase reporter containing a promoter with p65 binding site (see Supplementary Material, Fig. S9B) was provided by I. Quinto (45).

### Reagents

All the chemicals used in this study were dissolved in dimethyl sulfoxide (DMSO). Bafilomycin A1 was from Millipore; MG132; staurosporine and embelin were from Sigma. Primary antibodies used were: rabbit anti-XIAP, rabbit anti-Beclin 1, rabbit anti-Atg12 and anti-P-IκB Ser32/Ser36 (all diluted at 1:1000, Cell signaling), rabbit anti-actin and mouse anti-tubulin (both diluted at 1:4000, Sigma), rabbit anti-LC3 (diluted at 1:1000, Novus Biological), mouse anti-p62 (diluted at 1:1000, BD Bioscience), mouse anti-p53 and rabbit anti-NFκB p65 (diluted at 1:1000, Santa Cruz Biotechnology), mouse anti-HA (diluted at 1:2000, Covance). Anti-mouse and anti-rabbit HRP-conjugated secondary antibodies were from GE Healthcare. Propidium iodide, Alexa-Fluor-594- and Alexa-Fluor-488-conjugated antibodies were from Molecular Probes (Invitrogen).

### Western blot analysis

Cells were washed and harvested in ice-cold PBS and pellets were lysed on ice in RIPA buffer (150 mm NaCl, 1% Nonidet P-40, 0.5% sodium deoxycholate, 0.1% SDS, 50 mm Tris, pH 8.0) containing a protease/phosphatase inhibitors mix (Roche). After 1 h of incubation on ice with frequent agitations, cell lysates were centrifuged at 12 000 g, 10 min, the supernatants were collected and the concentration of proteins was determined using the DC Protein Assay, according to the manufacturer's instructions (Bio-Rad Laboratories). Proteins (25 μg) from the various lysates were separated on 10–16.5% polyacrylamide slab gels (depending on the size of the protein to be analysed) and transferred to polyvinylidene fluoride membranes. The membranes were blocked with 5% skimmed milk in PBS for 1 h at room temperature and reacted for 16 h at 4°C with the appropriate primary antibody. Primary and HRP-conjugated antibodies were applied in 3% BSA in PBS, containing 0.02% sodium azide. Incubations with secondary antibodies were for 1 h at room temperature. Membranes were rinsed between incubations three times with PBS plus 0.05% tween-20. After the last wash, membranes were imaged using ECL (GE Healthcare). Protein bands were quantified by densitometric analysis using ImageJ software.

### Fluorescence microscopy

Quantification of aggregate formation and LC3 dots was assessed as previously described (46). Two hundred EGFP-HDQ74-transfected cells were selected and the number of cells with aggregates was counted using a fluorescence microscope. The identity of the slides was unavailable to the observer until all slides had been studied.

For immunofluorescence staining, cells were cultured on coverslips, fixed with 3.7% paraformaldehyde in PBS for 10 min, permeabilized with 0.05% saponin in PBS for 10 min, blocked with 0.1% BSA in PBS for 10 min and incubated with primary antibodies overnight at 4°C. Cells were then washed three times with PBS and incubated with secondary Alexa-Fluor-conjugated antibodies. Both primary and secondary antibodies were prepared in 0.1% BSA in PBS. Samples were mounted using antifade reagent with DAPI (ProLong Gold; Invitrogen) and observed using a Zeiss Axiovert 200M microscope with an LSM 710 confocal atttachment, using a 63×1.4 numerical aperture Plan Apochromat oil-immersion lens. Automatic counting of LC3 vesicles from HeLa cells stably expressing GFP-mRFP-LC3 was performed using the Cellomics ArrayScan VTI HCS Reader (×40 objective) and the Spot Detector V3 Cellomics BioApplication (Thermo Fisher Scientific). Number of vesicles per cell was counted in 1000 cells per coverslip and the mean number of vesicles per cell was calculated by the ArrayScan software.

### Luciferase reporter assays

HeLa cells were seeded in six multiwells and transfected with 0.5 µg of the indicated luciferase reporter vectors plus 0.05 µg of the Renilla luciferase and cultured in a full medium for 24 h. Cells were then lysed in reporter lysis buffer (Promega). Firefly and Renilla luciferase activities were measured in a luminometer using the Dual-Glo luciferase assay kit (Promega). The relative luciferase activity (RLU) is defined as the firefly-to-Renilla luciferase activity ratio and normalized for the protein concentration of each sample.

### Transfections

For knockdown experiments, cells were transfected 72–96 h before analysis with a 50 nm final concentration of the indicated SMARTpool or deconvoluted siRNAs (Dharmacon) using lipofectamine 2000 (Invitrogen), according to the manufacturer's instructions. For overexpression experiments, cells were transfected with 1–2.5 µg of the respective constructs using *Trans*IT®-2020 Transfection Reagent (Mirus Bio LLC) according to the manufacturer's instructions.

### Quantitative real-time PCR

Total RNA was extracted from cells using Trizol (Invitrogen) and treated with Deoxyribunuclease I, Amplification Grade (Invitrogen). SuperScript III First-Strand Synthesis System (Invitrogen) and random hexadeoxynucleotide primers were used to synthesize cDNA. For the cDNA real-time PCR, the SYBR Green PCR master mix (AB applied Biosystem) was employed according to the manufacturer's instructions. The following sets of primers were used for the amplification of Beclin 1 cDNA: forward 5′-GCTCCATTACTTACCACAGC-3′ and reverse 5′-CAGTGACGTTGAGCTGAGTG-3′. The real-time PCR analyses were performed using 7900HT fast real-time PCR system (Applied Biosciences).

### Chromatin immunoprecipitation

10^8^ HeLa cells/condition were cross-linked using 1% formaldehyde in growth medium for 10 min and then cells were treated with 0.215 M Glycine for 5 min to stop the cross-linking and washed twice with PBS. Cells were lysed in buffer A (10 mm Tris pH 8.0, 10 mm NaCl, 0.2% NP40) supplemented with 10 mm NaBu and protease/phosphatase inhibitors mix (Roche) for 10 min on ice. The nuclei were recovered and resuspended in buffer B (50 mm Tris pH 8.1, 10 mm EDTA, 1% SDS) supplemented with 10 mm NaBu and protease/phosphatase inhibitors mix (Roche) and incubated for 10 min on ice. Cells were then diluted ×2 in buffer C (20 mm Tris pH 8.1, 2 mm EDTA, 150 mm NaCl, 1% Triton X100, 0.01% SDS) supplemented with 10 mm NaBu and protease/phosphatase inhibitors mix (Roche) before sonication for 10 min at 4°C. Chromatin was then cleared and equal amounts were incubated overnight at 4°C on a rotating wheel with anti-p65 antibody sc-372X (Santa Cruz Biotechnology), anti-Histone H3 (Abcam) and anti-mouse IgG produced in rabbit (Sigma). Immunocomplexes were isolated using protein A-sepharose (GE-Healthcare), washed twice with buffer D (20 mm Tris pH 8.1, 2 mm EDTA, 50 mm NaCl, 1% Triton X100, 0.1% SDS) and once with buffer E (10 mm Tris pH 8.1, 1 mm EDTA, 0.25 M LiCl, 1% NP-40, 0.1% sodium deoxycholate monohydrate) and finally once with TE buffer. Samples were then eluted using buffer F (100 mm NaHCO_3_, 1% SDS). The cross-linking was reversed by treating the sampled with RNase A and NaCl at a final concentration of 0.3 M overnight at 67° C and subsequent treatment with proteinase K (Fisher Scientific) for 2 h at 45°C.

Samples were then cleaned using Qiaquick PCR Purification Kit (Qiagen) and subjected to a real-time PCR analysis. The primers used for the amplification of p65 binding site in Beclin 1 promoter are: 5′-CCCGTATCATACCATTCCTAG-3′ and 5′-GAAACTCGTGTCCAGTTTCAG-3′ and for actin are: 5′-ATCTGGCACCACACCTTCT-3′ and 5′-TGGGGTGTTGAAGGTCTCA-3′.

### Cytometric analysis

After treatment, cells were stained with propidium iodide and FITC-conjugated Annexin A5 (Abcam). Subsequently, the emitted red (620 ± 20 nm band-pass filter) and green (488 ± 20 nm band-pass filter) fluorescence was analysed by flow cytometry. In each experiment, 10 000 cells per sample were collected and analysed using a Becton–Dickinson FACSCalibur 4-colour analyser.

### Statistical analysis

Densitometric analysis on the immunoblots was performed using Image J software. In all the main or supplementary Figures, error bars represent standard deviations. In all the experiments, *P*-values were determined by two-tailed Student's *t*-test or paired *t*-test for normalized control values, in a triplicate experiment representative of at least three independent experiments.

## Authors’ Contributions

F.L. and G.G. performed most of the experiments. S.L. and M.R. helped to plan the experiments. F.S. contributed preliminary data. D.C.R. supervised the studies, helped design and interpret experiments and helped write the paper with G.G. All authors discussed the results and commented on the manuscript.

## Supplementary Material

Supplementary Material is available at *HMG* online.

## Funding

We are grateful for funding from the Wellcome Trust (Principal Fellowship to D.C.R), NIHR Biomedical Research Unit in Dementia at Addenbrooke's Hospital, the Treat PolyQ project (European community's Seventh Framework Programme under grant agreement no. 264508), and the Jiangsu Government Scholarship for Overseas Studies. Funding to pay the Open Access publication charges for this article was provided by Wellcome Trust.

## Supplementary Material

Supplementary Data

## References

[1] Su M. Mei Y. Sinha S. (2013) Role of the crosstalk between autophagy and apoptosis in cancer. J. Oncol., 2013, 102735. 23840208 10.1155/2013/102735 PMC3687500

[2] Rubinsztein D.C. Codogno P. Levine B. (2012) Autophagy modulation as a potential therapeutic target for diverse diseases. Nat. Rev. Drug Discov., 11, 709–730. 22935804 10.1038/nrd3802 PMC3518431

[3] Rosenfeldt M.T. O'Prey J. Morton J.P. Nixon C. MacKay G. Mrowinska A. Au A. Rai T.S. Zheng L. Ridgway R. (2013) p53 status determines the role of autophagy in pancreatic tumour development. Nature, 504, 296–300. 24305049 10.1038/nature12865

[4] Morselli E. Galluzzi L. Kepp O. Vicencio J.M. Criollo A. Maiuri M.C. Kroemer G. (2009) Anti- and pro-tumor functions of autophagy. Biochim. Biophys. Acta, 1793, 1524–1532. 19371598 10.1016/j.bbamcr.2009.01.006

[5] Qu X. Yu J. Bhagat G. Furuya N. Hibshoosh H. Troxel A. Rosen J. Eskelinen E.L. Mizushima N. Ohsumi Y. (2003) Promotion of tumorigenesis by heterozygous disruption of the beclin 1 autophagy gene. J. Clin. Invest., 112, 1809–1820. 14638851 10.1172/JCI20039 PMC297002

[6] Yue Z. Jin S. Yang C. Levine A.J. Heintz N. (2003) Beclin 1, an autophagy gene essential for early embryonic development, is a haploinsufficient tumor suppressor. Proc. Natl Acad. Sci. USA, 100, 15077–15082. 14657337 10.1073/pnas.2436255100 PMC299911

[7] Chen H.Y. White E. (2011) Role of autophagy in cancer prevention. Cancer Prev. Res. (Phila), 4, 973–983. 21733821 10.1158/1940-6207.CAPR-10-0387 PMC3136921

[8] Silke J. Meier P. (2013) Inhibitor of apoptosis (IAP) proteins-modulators of cell death and inflammation. Cold Spring Harb. Perspect. Biol., 5 doi:10.1101/cshperspect.a008730. 10.1101/cshperspect.a008730 PMC3552501 23378585

[9] Yang Y. Fang S. Jensen J.P. Weissman A.M. Ashwell J.D. (2000) Ubiquitin protein ligase activity of IAPs and their degradation in proteasomes in response to apoptotic stimuli. Science, 288, 874–877. 10797013 10.1126/science.288.5467.874

[10] Gyrd-Hansen M. Darding M. Miasari M. Santoro M.M. Zender L. Xue W. Tenev T. da Fonseca P.C. Zvelebil M. Bujnicki J.M. (2008) IAPs contain an evolutionarily conserved ubiquitin-binding domain that regulates NF-kappaB as well as cell survival and oncogenesis. Nat. Cell. Biol., 10, 1309–1317. 18931663 10.1038/ncb1789 PMC2818601

[11] Fulda S. (2012) Inhibitor of Apoptosis (IAP) proteins as therapeutic targets for radiosensitization of human cancers. Cancer Treat. Rev., 38, 760–766. 22342104 10.1016/j.ctrv.2012.01.005

[12] Jaffer S. Orta L. Sunkara S. Sabo E. Burstein D.E. (2007) Immunohistochemical detection of antiapoptotic protein X-linked inhibitor of apoptosis in mammary carcinoma. Hum. Pathol., 38, 864–870. 17350670 10.1016/j.humpath.2006.11.016

[13] Kluger H.M. McCarthy M.M. Alvero A.B. Sznol M. Ariyan S. Camp R.L. Rimm D.L. Mor G. (2007) The X-linked inhibitor of apoptosis protein (XIAP) is up-regulated in metastatic melanoma, and XIAP cleavage by Phenoxodiol is associated with Carboplatin sensitization. J. Transl. Med., 5, 6. 17257402 10.1186/1479-5876-5-6 PMC1796544

[14] Ramp U. Krieg T. Caliskan E. Mahotka C. Ebert T. Willers R. Gabbert H.E. Gerharz C.D. (2004) XIAP expression is an independent prognostic marker in clear-cell renal carcinomas. Hum. Pathol., 35, 1022–1028. 15297970 10.1016/j.humpath.2004.03.011

[15] Parton M. Krajewski S. Smith I. Krajewska M. Archer C. Naito M. Ahern R. Reed J. Dowsett M. (2002) Coordinate expression of apoptosis-associated proteins in human breast cancer before and during chemotherapy. Clin. Cancer Res., 8, 2100–2108. 12114409

[16] Hofmann H.S. Simm A. Hammer A. Silber R.E. Bartling B. (2002) Expression of inhibitors of apoptosis (IAP) proteins in non-small cell human lung cancer. J. Cancer Res. Clin. Oncol., 128, 554–560. 12384799 10.1007/s00432-002-0364-z PMC12164409

[17] Liu S. Zhang P. Chen Z. Liu M. Li X. Tang H. (2013) MicroRNA-7 downregulates XIAP expression to suppress cell growth and promote apoptosis in cervical cancer cells. FEBS Lett., 587, 2247–2253. 23742934 10.1016/j.febslet.2013.05.054

[18] Eschenburg G. Eggert A. Schramm A. Lode H.N. Hundsdoerfer P. (2012) Smac mimetic LBW242 sensitizes XIAP-overexpressing neuroblastoma cells for TNF-alpha-independent apoptosis. Cancer Res., 72, 2645–2656. 22491673 10.1158/0008-5472.CAN-11-4072

[19] Fulda S. (2012) Exploiting inhibitor of apoptosis proteins as therapeutic targets in hematological malignancies. Leukemia, 26, 1155–1165. 22230799 10.1038/leu.2012.4

[20] Tamm I. Kornblau S.M. Segall H. Krajewski S. Welsh K. Kitada S. Scudiero D.A. Tudor G. Qui Y.H. Monks A. (2000) Expression and prognostic significance of IAP-family genes in human cancers and myeloid leukemias. Clin. Cancer Res., 6, 1796–1803. 10815900

[21] Kabeya Y. Mizushima N. Ueno T. Yamamoto A. Kirisako T. Noda T. Kominami E. Ohsumi Y. Yoshimori T. (2000) LC3, a mammalian homologue of yeast Apg8p, is localized in autophagosome membranes after processing. EMBO J., 19, 5720–5728. 11060023 10.1093/emboj/19.21.5720 PMC305793

[22] Menzies F.M. Moreau K. Puri C. Renna M. Rubinsztein D.C. (2012) Measurement of autophagic activity in mammalian cells. Curr. Protoc. Cell. Biol., Chapter 15, Unit 15 16. 10.1002/0471143030.cb1516s54 22422474

[23] Klionsky D.J. Abdalla F.C. Abeliovich H. Abraham R.T. Acevedo-Arozena A. Adeli K. Agholme L. Agnello M. Agostinis P. Aguirre-Ghiso J.A. (2012) Guidelines for the use and interpretation of assays for monitoring autophagy. Autophagy, 8, 445–544. 22966490 10.4161/auto.19496 PMC3404883

[24] Kimura S. Noda T. Yoshimori T. (2007) Dissection of the autophagosome maturation process by a novel reporter protein, tandem fluorescent-tagged LC3. Autophagy, 3, 452–460. 17534139 10.4161/auto.4451

[25] Nikolovska-Coleska Z. Xu L. Hu Z. Tomita Y. Li P. Roller P.P. Wang R. Fang X. Guo R. Zhang M. (2004) Discovery of embelin as a cell-permeable, small-molecular weight inhibitor of XIAP through structure-based computational screening of a traditional herbal medicine three-dimensional structure database. J. Med. Chem., 47, 2430–2440. 15115387 10.1021/jm030420+

[26] Ahn K.S. Sethi G. Aggarwal B.B. (2007) Embelin, an inhibitor of X chromosome-linked inhibitor-of-apoptosis protein, blocks nuclear factor-kappaB (NF-kappaB) signaling pathway leading to suppression of NF-kappaB-regulated antiapoptotic and metastatic gene products. Mol. Pharmacol., 71, 209–219. 17028156 10.1124/mol.106.028787

[27] Korolchuk V.I. Mansilla A. Menzies F.M. Rubinsztein D.C. (2009) Autophagy inhibition compromises degradation of ubiquitin-proteasome pathway substrates. Mol. Cell, 33, 517–527. 19250912 10.1016/j.molcel.2009.01.021 PMC2669153

[28] Ravikumar B. Duden R. Rubinsztein D.C. (2002) Aggregate-prone proteins with polyglutamine and polyalanine expansions are degraded by autophagy. Hum. Mol. Genet, 11, 1107–1117. 11978769 10.1093/hmg/11.9.1107

[29] Axe E.L. Walker S.A. Manifava M. Chandra P. Roderick H.L. Habermann A. Griffiths G. Ktistakis N.T. (2008) Autophagosome formation from membrane compartments enriched in phosphatidylinositol 3-phosphate and dynamically connected to the endoplasmic reticulum. J. Cell Biol., 182, 685–701. 18725538 10.1083/jcb.200803137 PMC2518708

[30] Copetti T. Bertoli C. Dalla E. Demarchi F. Schneider C. (2009) p65/RelA modulates BECN1 transcription and autophagy. Mol. Cell Biol., 29, 2594–2608. 19289499 10.1128/MCB.01396-08 PMC2682036

[31] Ravikumar B. Imarisio S. Sarkar S. O'Kane C.J. Rubinsztein D.C. (2008) Rab5 modulates aggregation and toxicity of mutant huntingtin through macroautophagy in cell and fly models of Huntington disease. J. Cell Sci., 121, 1649–1660. 18430781 10.1242/jcs.025726 PMC2635563

[32] Lu M. Lin S.C. Huang Y. Kang Y.J. Rich R. Lo Y.C. Myszka D. Han J. Wu H. (2007) XIAP induces NF-kappaB activation via the BIR1/TAB1 interaction and BIR1 dimerization. Mol. Cell, 26, 689–702. 17560374 10.1016/j.molcel.2007.05.006 PMC1991276

[33] Freudlsperger C. Bian Y. Contag Wise S. Burnett J. Coupar J. Yang X. Chen Z. Van Waes C. (2013) TGF-beta and NF-kappaB signal pathway cross-talk is mediated through TAK1 and SMAD7 in a subset of head and neck cancers. Oncogene, 32, 1549–1559. 22641218 10.1038/onc.2012.171 PMC3434281

[34] Oeckinghaus A. Ghosh S. (2009) The NF-kappaB family of transcription factors and its regulation. Cold Spring Harb. Perspect. Biol., 1, a000034. 20066092 10.1101/cshperspect.a000034 PMC2773619

[35] Hussain A.R. Uddin S. Ahmed M. Bu R. Ahmed S.O. Abubaker J. Sultana M. Ajarim D. Al-Dayel F. Bavi P.P. (2010) Prognostic significance of XIAP expression in DLBCL and effect of its inhibition on AKT signalling. J. Pathol., 222, 180–190. 20632385 10.1002/path.2747

[36] Gyrd-Hansen M. Meier P. (2010) IAPs: from caspase inhibitors to modulators of NF-kappaB, inflammation and cancer. Nat. Rev. Cancer, 10, 561–574. 20651737 10.1038/nrc2889

[37] Sinha S. Levine B. (2008) The autophagy effector Beclin 1: a novel BH3-only protein. Oncogene, 27(Suppl. 1), S137–S148. 19641499 10.1038/onc.2009.51 PMC2731580

[38] Huang X. Wu Z. Mei Y. Wu M. (2013) XIAP inhibits autophagy via XIAP-Mdm2-p53 signalling. EMBO J., 32, 2204–2216. 23749209 10.1038/emboj.2013.133 PMC3746193

[39] LaCasse E.C. Mahoney D.J. Cheung H.H. Plenchette S. Baird S. Korneluk R.G. (2008) IAP-targeted therapies for cancer. Oncogene, 27, 6252–6275. 18931692 10.1038/onc.2008.302

[40] Petersen S.L. Wang L. Yalcin-Chin A. Li L. Peyton M. Minna J. Harran P. Wang X. (2007) Autocrine TNFalpha signaling renders human cancer cells susceptible to Smac-mimetic-induced apoptosis. Cancer Cell, 12, 445–456. 17996648 10.1016/j.ccr.2007.08.029 PMC3431210

[41] White E. (2012) Deconvoluting the context-dependent role for autophagy in cancer. Nat. Rev. Cancer, 12, 401–410. 22534666 10.1038/nrc3262 PMC3664381

[42] Laddha S.V. Ganesan S. Chan C.S. White E. (2014) Mutational landscape of the essential autophagy gene BECN1 in human cancers. Mol. Cancer Res., 12, 485–490. 24478461 10.1158/1541-7786.MCR-13-0614 PMC3989371

[43] Yang A. Rajeshkumar N.V. Wang X. Yabuuchi S. Alexander B.M. Chu G.C. Von Hoff D.D. Maitra A. Kimmelman A.C. (2014) Autophagy is critical for pancreatic tumor growth and progression in tumors with p53 alterations. Cancer Discov., 4, 905–913. 24875860 10.1158/2159-8290.CD-14-0362 PMC4125497

[44] Csomos R.A. Wright C.W. Galban S. Oetjen K.A. Duckett C.S. (2009) Two distinct signalling cascades target the NF-kappaB regulatory factor c-IAP1 for degradation. Biochem. J., 420, 83–91. 19243308 10.1042/BJ20082140 PMC2677214

[45] Puca A. Fiume G. Palmieri C. Trimboli F. Olimpico F. Scala G. Quinto I. (2007) IkappaB-alpha represses the transcriptional activity of the HIV-1 Tat transactivator by promoting its nuclear export. J. Biol. Chem., 282, 37146–37157. 17942396 10.1074/jbc.M705815200

[46] Sarkar S. Krishna G. Imarisio S. Saiki S. O'Kane C.J. Rubinsztein D.C. (2008) A rational mechanism for combination treatment of Huntington's disease using lithium and rapamycin. Hum. Mol.Genet., 17, 170–178. 17921520 10.1093/hmg/ddm294

