# Supplementary material for: XIAP and cIAP1 amplifications induce Beclin 1-dependent autophagy through NFκB activation
Source: Hum Mol Genet. 2015 Feb 10;24(10):2899–913. doi: 10.1093/hmg/ddv052 (PMC4406300; doi:10.1093/hmg/ddv052)
Supplement: Supplementary Data [file supp_24_10_2899__index.html]

XIAP and cIAP1 amplifications induce Beclin 1-dependent autophagy through NFκB activation — XIAP and cIAP1 amplifications induce Beclin 1-dependent autophagy through NFκB activation — XIAP and cIAP1 amplifications induce Beclin 1-dependent autophagy through NFκB activation — Supplementary Data 

# XIAP and cIAP1 amplifications induce Beclin 1-dependent autophagy through NFκB activation

## Supplementary Data

Supplementary Data

**Files in this Data Supplement:**

- Supplementary Data - Pdf file
