# Supplementary material for: XIAP and cIAP1 amplifications induce Beclin 1-dependent autophagy through NFκB activation
Source: Hum Mol Genet. 2015 Feb 10;24(10):2899–913. doi: 10.1093/hmg/ddv052 (PMC4406300; doi:10.1093/hmg/ddv052)
Supplement: Supplementary Data [file supp_ddv052_ddv052supp.pdf]

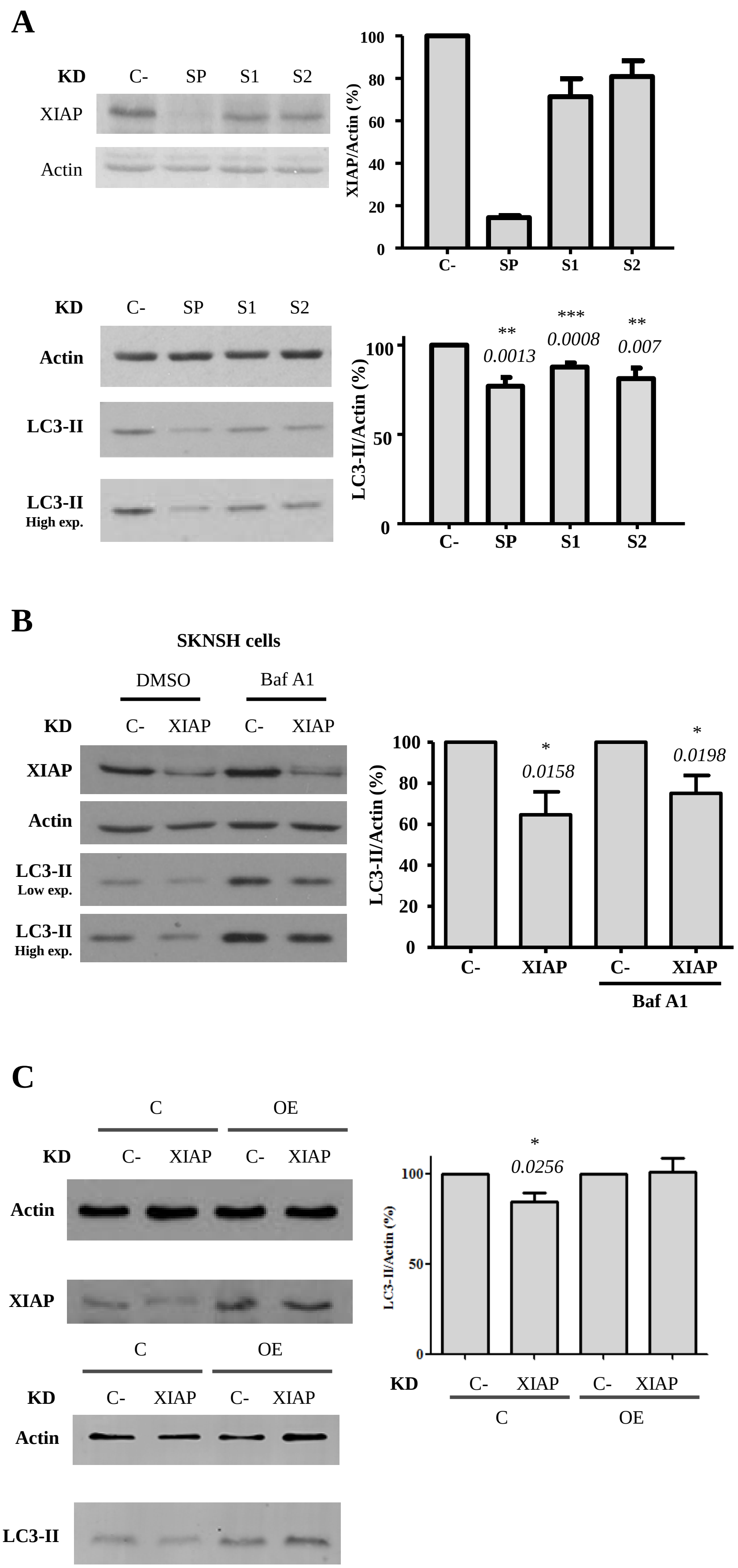
A
KD C- SP S1 S2
XIAP
Actin
XIAP/Actin (%)
100
80
60
40
20
0
C- SP S1 S2
KD C- SP S1 S2
Actin
LC3-II
LC3-II High exp.
LC3-II/Actin (%)
** 0.0013
*** 0.0008
** 0.007
100
50
0
C- SP S1 S2
B
SKNSH cells
DMSO
Baf A1
KD C- XIAP C- XIAP
XIAP
Actin
LC3-II Low exp.
LC3-II High exp.
LC3-II/Actin (%)
* 0.0158
* 0.0198
100
80
60
40
20
0
C- XIAP C- XIAP
Baf A1
C
C OE
KD C- XIAP C- XIAP
Actin
XIAP
C OE
KD C- XIAP C- XIAP
Actin
LC3-II
LC3-II/Actin (%)
* 0.0256
100
50
0
KD C- XIAP C- XIAP
C OE

Fig. S1

Supplementary Fig. 1: XIAP induces autophagy. (A) (Top panel) HeLa cells were transfected with a control (C-), a smartpool (SP) or deconvoluted XIAP siRNAs (S1 and S2) for 72 h. Next, the cells were lysed and analysed by western blotting. The efficiency of XIAP knockdown is shown in the graph on the right. (Bottom panel) HeLa cells were transfected as in the top panel and were treated with 400 nM bafilomycin A1 during the last 4 h. Densitometric measurements of LC3-II bands were normalised to the corresponding actin bands and are shown in the histogram on the right. **(B)** SKNSH human neuroblastoma cells were transfected with a control (C-) or a smartpool XIAP siRNA for 96 h. During the last 4h, cells were treated with DMSO or 400 nM bafilomycin A1 (Baf A1) and were subsequently subjected to western blotting. The western blot is representative of the efficiency of XIAP knockdown (KD) and of the levels of LC3-II in these conditions. The controls in lanes 1 and 3 are both set at 100%. **(C)** MCF10A cells were transfected with a control (C-) or XIAP siRNA for 96h. In the last 48 h, cells were transfected with empty vector (C-) or XIAP (OE) expression constructs. Densitometric measurements of LC3-II bands were normalised to the corresponding actin bands and are shown in the histogram on the right. The values shown in all the histograms represent the mean+standard deviation from at least three independent experiments performed in triplicate samples/condition. The controls in lanes 1 and 3 are both set at 100%. The *P*-values were determined using Student's t-test.

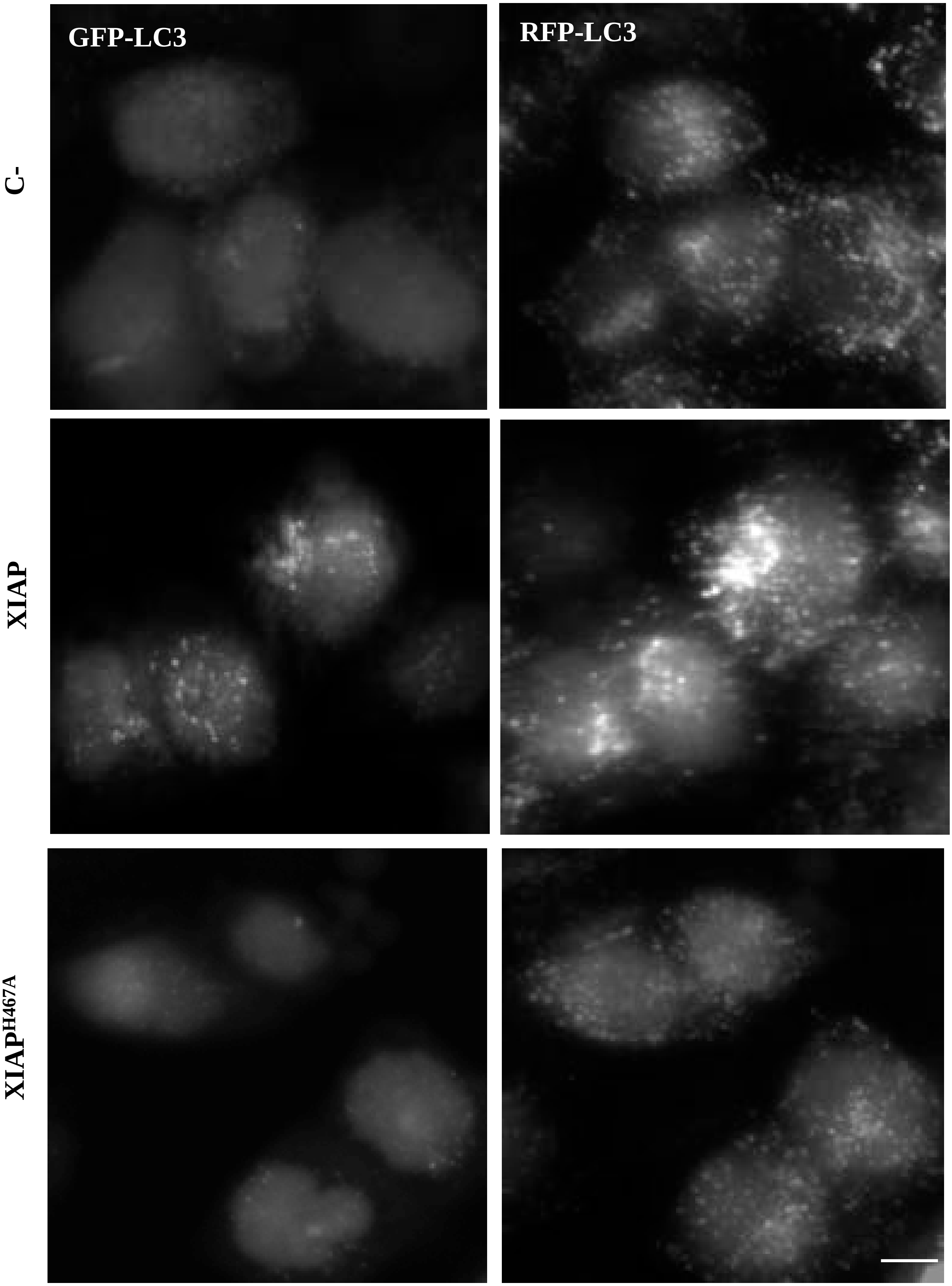

Fig. S2

Supplementary Fig. 2: Representative images for GFP-mRFP-LC3. HeLa cells stably expressing mRFP-GFP-LC3 transfected with empty vector (C-) or XIAP expression constructs for 48 h were fixed and imaged using the Cellomics ArrayScan VTI HCS Reader (40x objective) and the Spot Detector V3 Cellomics BioApplication (Thermo Fisher Scientific). Note that these are not confocal images but were the images that were obtained from the automated microcope. Bar, 10 µm.

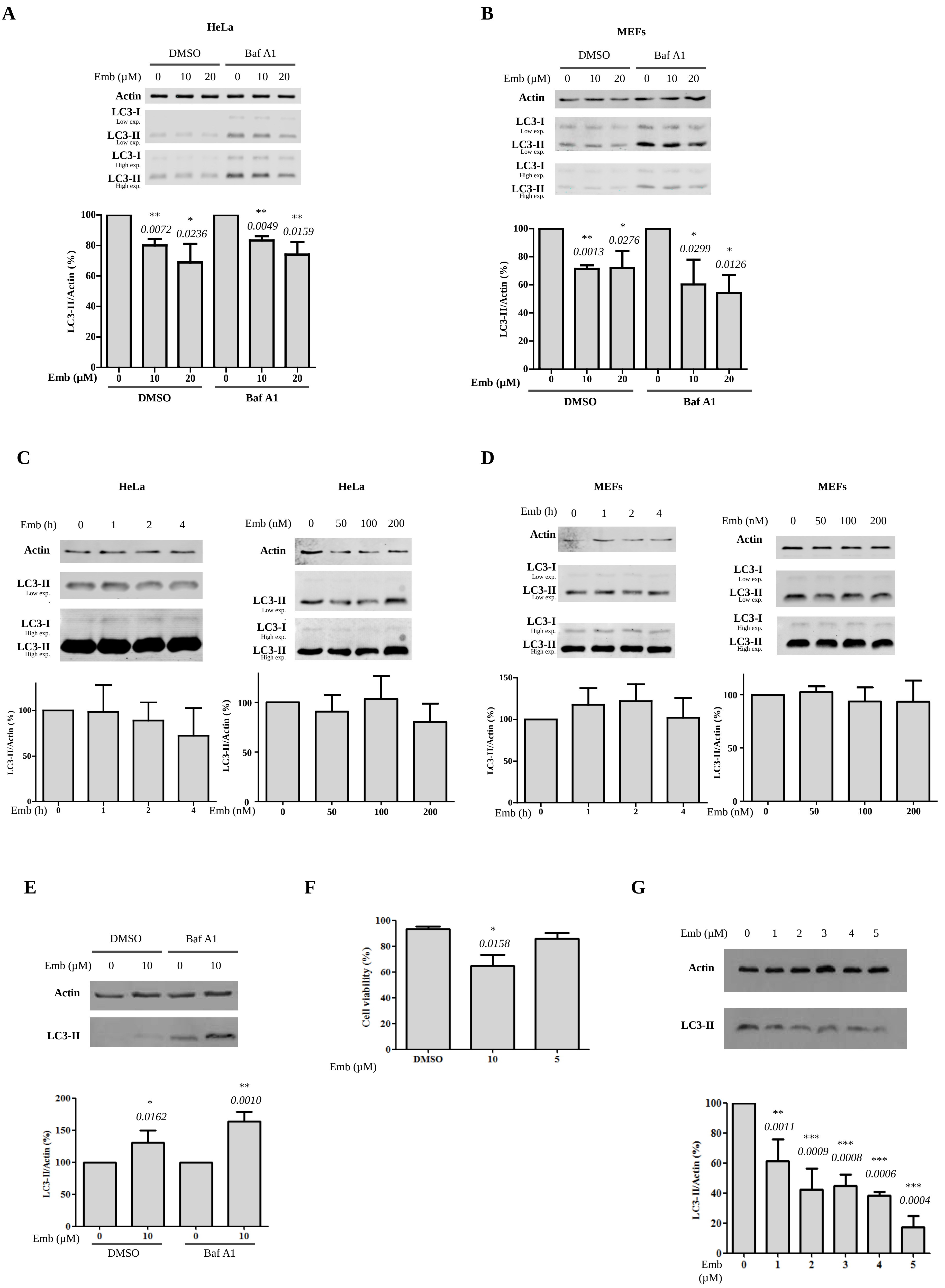
A
HeLa
DMSO
Baf A1
Emb (μM) 0 10 20 0 10 20
Actin
LC3-I Low exp.
LC3-II Low exp.
LC3-I High exp.
LC3-II High exp.
LC3-II/Actin (%)
** 0.0072
* 0.0236
** 0.0049
** 0.0159
Emb (μM) 0 10 20 0 10 20
DMSO
Baf A1
B
MEFs
DMSO
Baf A1
Emb (μM) 0 10 20 0 10 20
Actin
LC3-I Low exp.
LC3-II Low exp.
LC3-I High exp.
LC3-II High exp.
LC3-II/Actin (%)
** 0.0013
* 0.0276
* 0.0299
* 0.0126
Emb (μM) 0 10 20 0 10 20
DMSO
Baf A1
C
HeLa
Emb (h) 0 1 2 4
Actin
LC3-II Low exp.
LC3-I High exp.
LC3-II High exp.
HeLa
Emb (nM) 0 50 100 200
Actin
LC3-II Low exp.
LC3-I High exp.
LC3-II High exp.
LC3-II/Actin (%)
Emb (h) 0 1 2 4
Emb (nM) 0 50 100 200
D
MEFs
Emb (h) 0 1 2 4
Actin
LC3-I Low exp.
LC3-II Low exp.
LC3-I High exp.
LC3-II High exp.
MEFs
Emb (nM) 0 50 100 200
Actin
LC3-I Low exp.
LC3-II Low exp.
LC3-I High exp.
LC3-II High exp.
LC3-II/Actin (%)
Emb (h) 0 1 2 4
Emb (nM) 0 50 100 200
E
DMSO
Baf A1
Emb (μM) 0 10 0 10
Actin
LC3-II
LC3-II/Actin (%)
* 0.0162
** 0.0010
Emb (μM) 0 10 0 10
DMSO
Baf A1
F
Cell viability (%)
* 0.0158
Emb (μM) DMSO 10 5
G
Emb (μM) 0 1 2 3 4 5
Actin
LC3-II
LC3-II/Actin (%)
** 0.0011
*** 0.0009
*** 0.0008
*** 0.0006
*** 0.0004
Emb (μM) 0 1 2 3 4 5

Fig. S3

Supplementary Fig. 3:Embelin effect on autophagy. HeLa cells **(A)** and **(B)** mouse embryonic fibroblasts were treated for 16 h with DMSO or with 10 µM or 20 µM embelin (Emb). During the last 4 h, cells were treated with DMSO or 400 nM bafilomycin A1 (Baf A1) and were subsequently subjected to western blotting. The western blot is representative of the levels of LC3-II in these conditions. **(C)** HeLa cells and mouse embryonic fibroblasts **(D)** were treated with 100 nM embelin (Emb) for 0, 1, 2 or 4 h (All left panels) and for 4 h with DMSO or with 50, 100, or 200 nM embelin (Emb) (All right panels). **(E)** MCF10A cells were treated for 16 h with DMSO or with 10 µM embelin (Emb). During the last 4 h, cells were treated with DMSO or 400 nM bafilomycin A1 (Baf A1) and were subsequently subjected to western blotting. The western blot is representative of the levels of LC3-II in these conditions. Densitometric measurements of LC3-II bands were normalised to the corresponding actin bands and are shown in the histogram on the right. **(F)** MCF10A cells were treated for 16 h with DMSO or with 10 or 5 µM embelin (Emb). Then cells were subjected to trypan blue staining and trypan blue positive cells were counted. **(G)** MCF10A cells were treated for 16 h with DMSO or with 1, 2, 3, 4 or 5 µM embelin (Emb). During the last 4 h, cells were treated with DMSO or 400 nM bafilomycin A1 (Baf A1) and were subsequently subjected to western blotting. The western blot is representative of the levels of LC3-II in these conditions. Densitometric measurements of LC3-II bands were normalised to the corresponding actin bands and are shown in the histogram on the right.

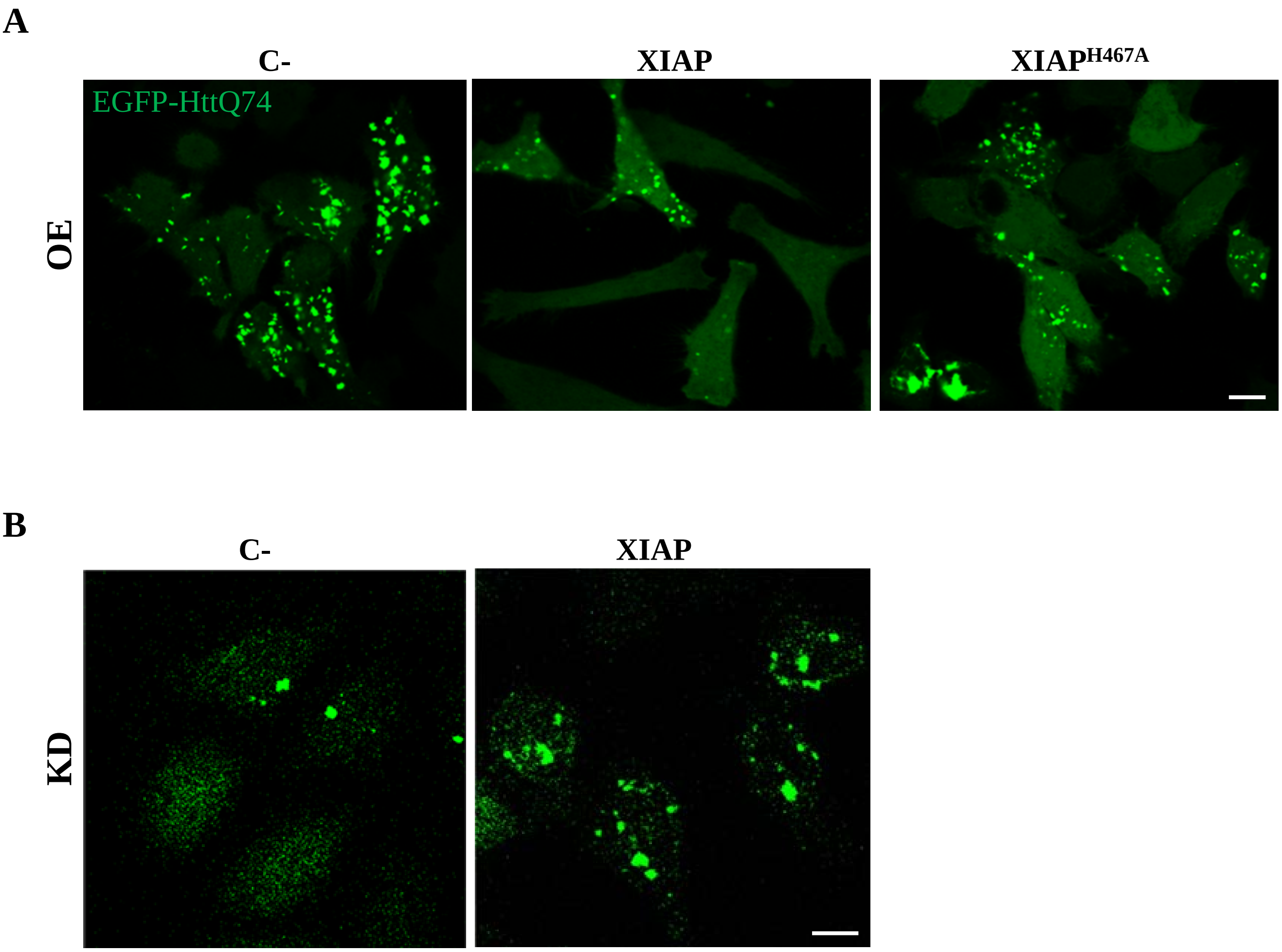

Fig. S4

Supplementary Fig. 4: Representative images for GFP-mRFP-LC3 dots and EGFP-HttQ74 aggregates under cIAP1 overexpression. HeLa cells were co-transfected with the GFP-HttQ74 expression construct plus empty vector (C-) or XIAP expression constructs for 48 h (upper panel) **(A)** or with a control (C-) or XIAP siRNAs **(B)** and 24 h later, the cells were transfected with the GFP-HttQ74 expression construct for 48 h (lower panel). In both panels, the cells were fixed and imaged using a confocal microscope. Bars, 10 µm.

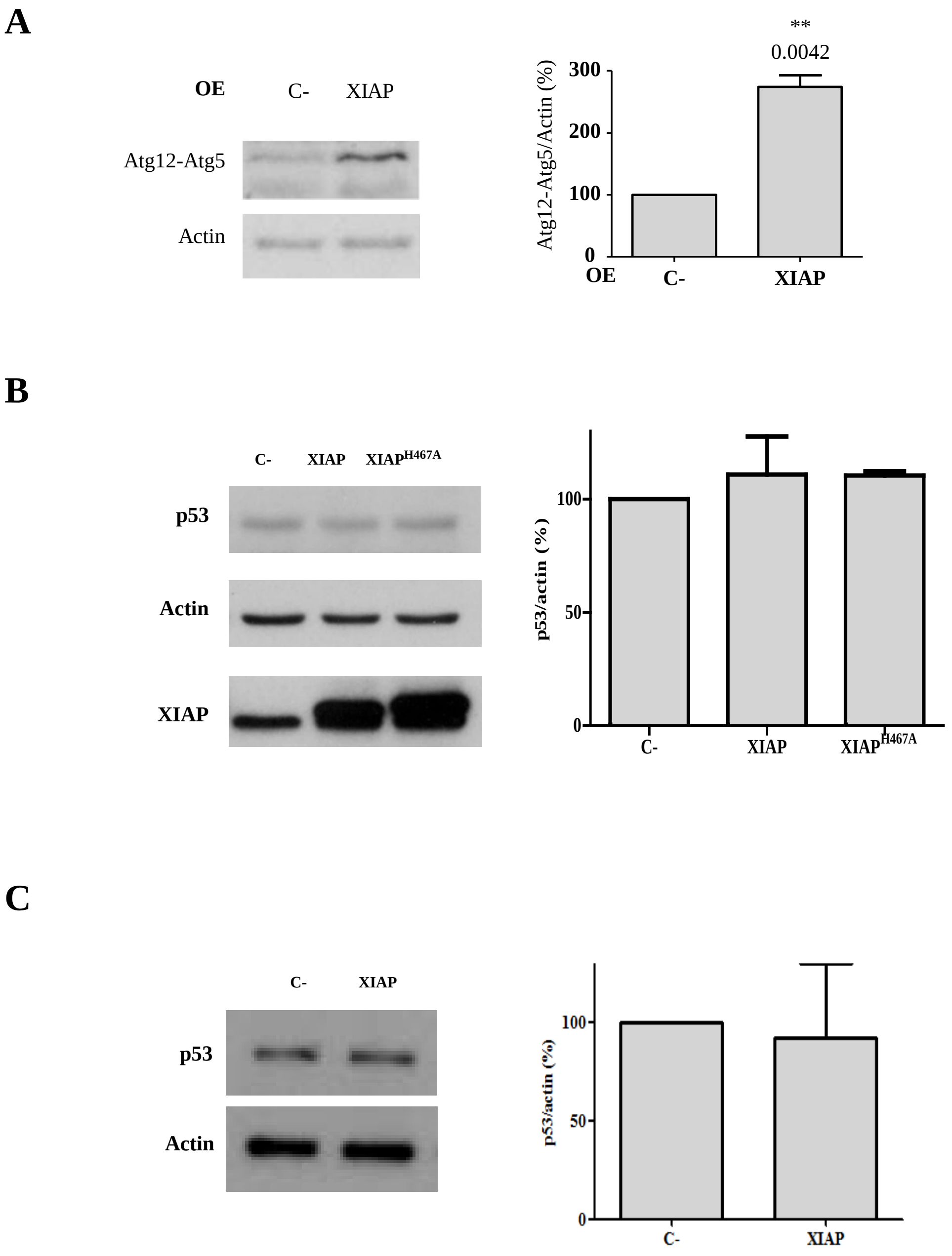
A
OE
C-
XIAP
Atg12-Atg5
Actin
**
0.0042
Atg12-Atg5/Actin (%)
300
200
100
0
OE
C-
XIAP
B
C-
XIAP
XIAP^H467A
p53
Actin
XIAP
p53/actin (%)
100
50
0
C-
XIAP
XIAP^H467A
C
C-
XIAP
p53
Actin
p53/actin (%)
100
50
0
C-
XIAP

Fig. S5

Supplementary Fig. 5: XIAP up-regulates Atg12-Atg5 conjugation. (A) HeLa cells were transfected with empty vector (C-) or XIAP expression constructs for 48 h. Densitometric measurements of Atg12-Atg5 conjugate bands were normalised to the corresponding actin bands and are shown in the histogram on the bottom. **(B)** HeLa cells previously transfected with empty vector (C-), wild type XIAP or $XIAP^{H467A}$ expression constructs for 48 h were subjected to western blotting. Densitometric measurements of p53 bands were normalised to the corresponding bands of actin and are shown in the histogram on the right. **(C)** MCF10A cells were transfected with empty vector (C-) or XIAP (XIAP) expression constructs. The values shown in all the histograms represent the mean+standard deviation from at least three independent experiments. The *P*-values were determined using Student's t-test.

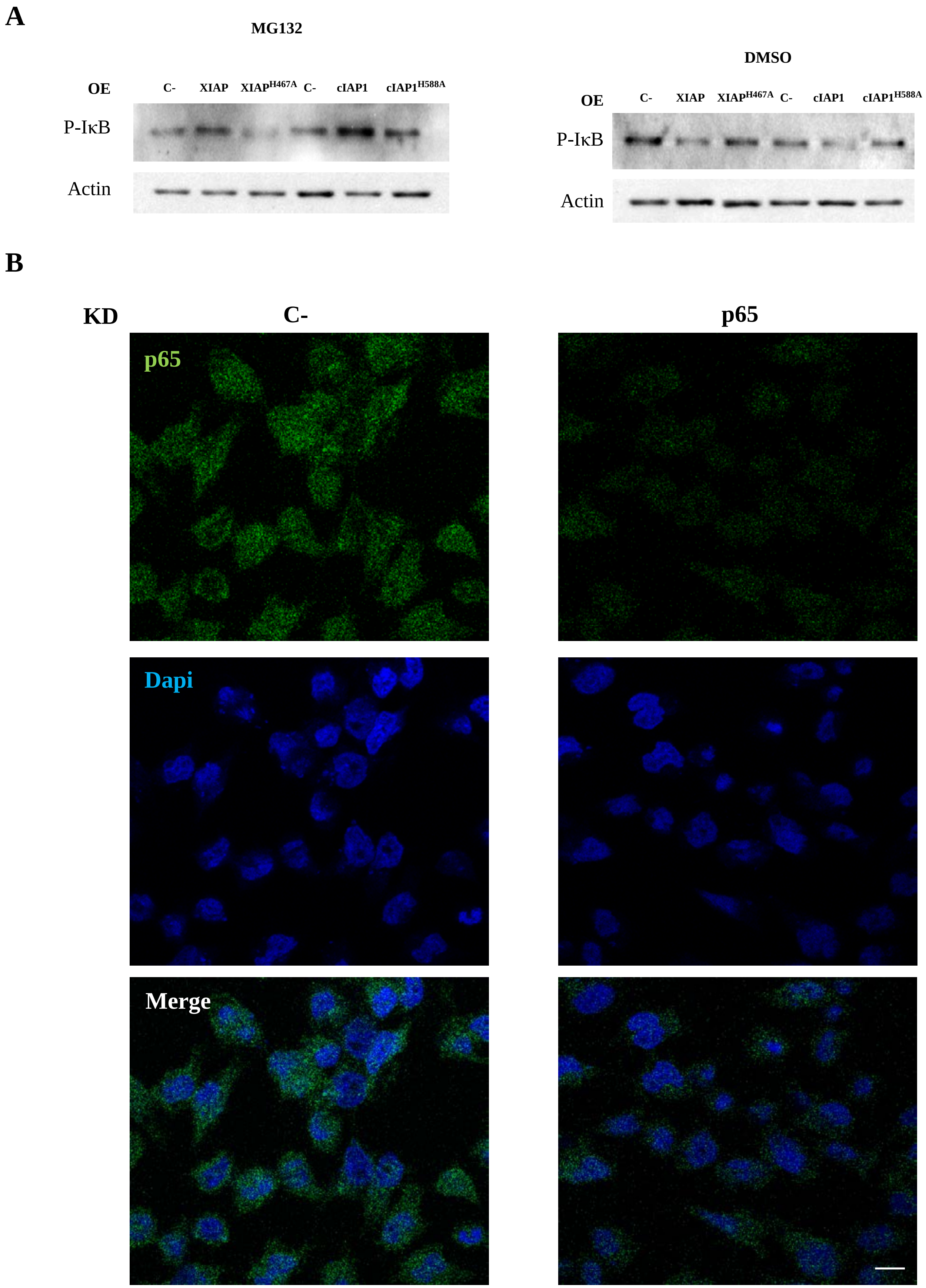
A
MG132
OE
C-
XIAP
XIAPH467A
C-
cIAP1
cIAP1H588A
P-IκB
Actin
DMSO
OE
C-
XIAP
XIAPH467A
C-
cIAP1
cIAP1H588A
P-IκB
Actin
B
KD
C-
p65
p65
Dapi
Merge

Fig. S6

Supplementary Fig. 6: XIAP and CIAP1 influence P-IκB levels. (A) HeLa cells previously transfected with empty vector (C-), wild type XIAP or $XIAP_{H467A}$ (three first lanes) and with empty vector (C-), wild type CIAP1 or $CIAP1_{H588A}$ expression (three second lanes) for 48 h were treated with 50 μM MG132 (left panel) or DMSO (right panel) during the last 4 h. **(B)** HeLa cells were transfected with a control (C-) or p65 siRNA for 72h and were then fixed and immunostained with p65 antibody. Bar, 10 μm.

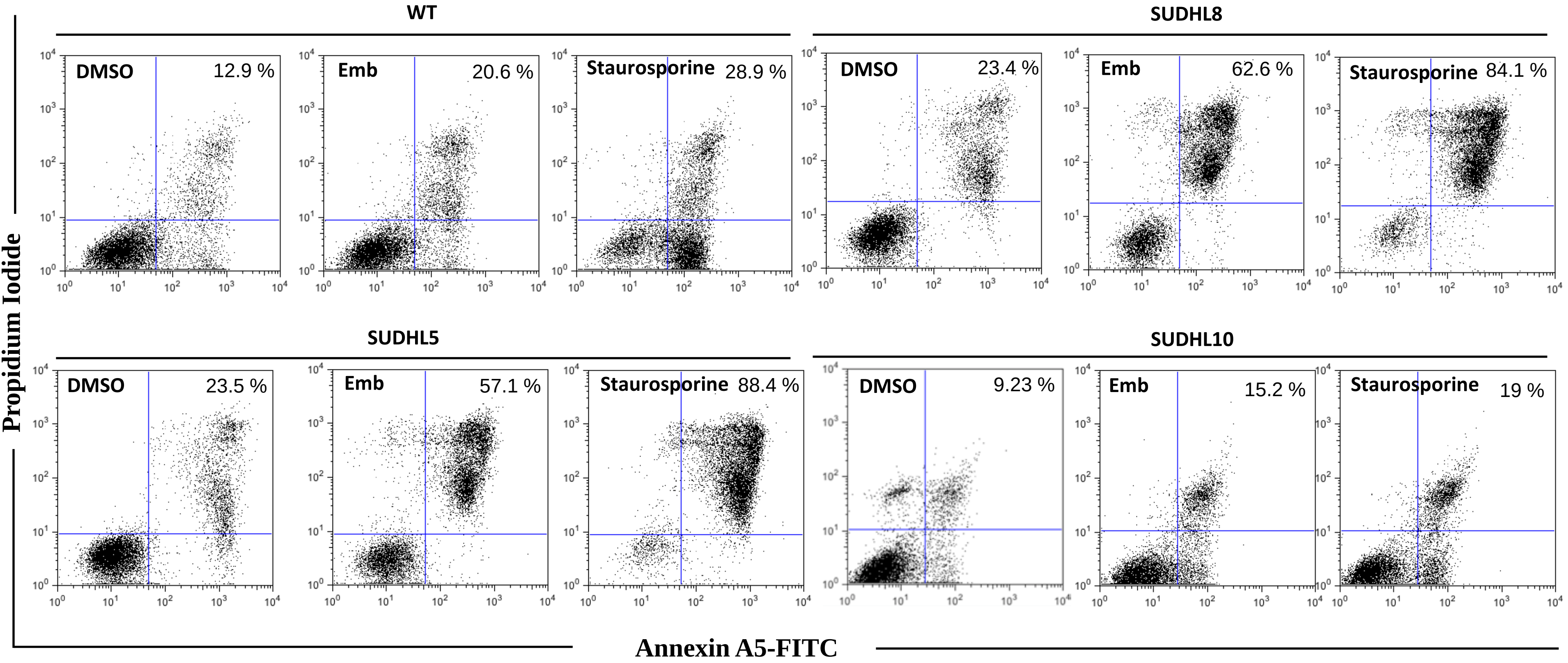
WT
SUDHL8
SUDHL5
SUDHL10
DMSO
12.9 %
Emb
20.6 %
Staurosporine
28.9 %
DMSO
23.4 %
Emb
62.6 %
Staurosporine
84.1 %
DMSO
23.5 %
Emb
57.1 %
Staurosporine
88.4 %
DMSO
9.23 %
Emb
15.2 %
Staurosporine
19 %
Propidium Iodide
Annexin A5-FITC

Fig. S7

Supplementary Fig. 7: XIAP inhibition induces apoptosis in B-cell lymphoma. B cells (WT) and three cell lines of diffuse large B-cell lymphoma, SUDHL5, SUDHL8 and SUDHL10 were were treated without or with 10 μM embelin (Emb) or 1 μM staurosporine for 16 h and were subsequently stained with Propidium iodide and FITC-conjugated Annexin A5. The percentage of the stained cells was detected by flow cytometry. The histograms are representative examples of data from three independent experiments performed in triplicate.

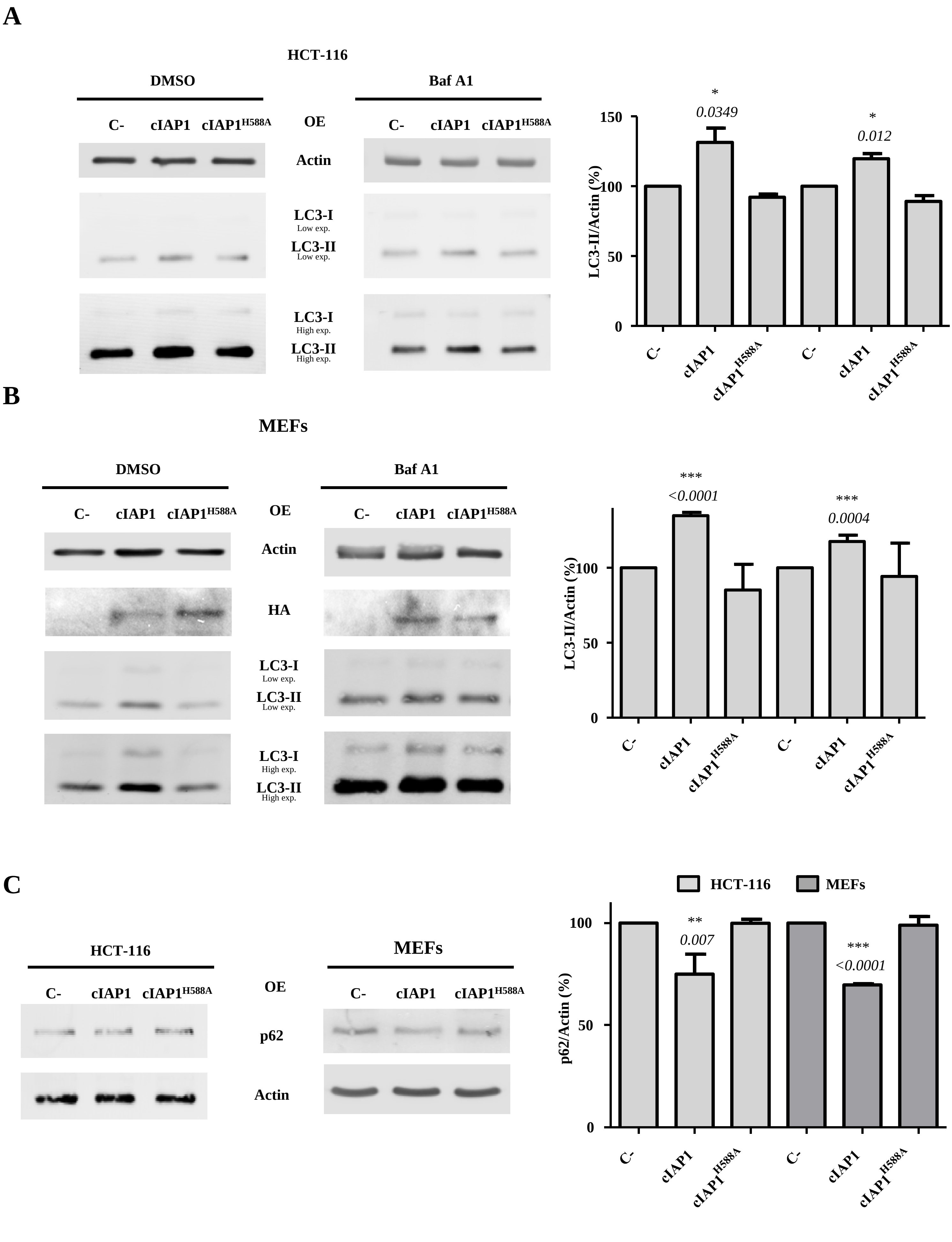
A
HCT-116
DMSO
Baf A1
C-
cIAP1
cIAP1H588A
OE
Actin
LC3-I
Low exp.
LC3-II
Low exp.
LC3-I
High exp.
LC3-II
High exp.
LC3-II/Actin (%)
150
100
50
0
*
0.0349
*
0.012
B
MEFs
HA
LC3-II/Actin (%)
***
<0.0001
***
0.0004
C
HCT-116
MEFs
p62
p62/Actin (%)
**
0.007
***
<0.0001

Fig. S8

Supplementary Fig. 8: CIAP1 overexpression induces autophagy. HCT-116 cells **(A)** and mouse embryonic fibroblasts **(B)** were transfected with empty vector (C-), wild type CIAP1 or $CIAP1_{H588A}$ expression constructs for 48 h and were treated with DMSO or 400 nM bafilomycin A1 (Baf A1) during the last 4 h. Densitometric measurements of LC3-II bands were normalised to the corresponding bands of actin and are shown in the histograms on the right. **(C)** HCT-116 cells and mouse embryonic fibroblasts were transfected as in (A) and (B) and were subjected to western blotting. Densitometric measurements of p62 bands were normalised to the corresponding bands of actin and are shown in the histogram on the right. The values shown in all the histograms represent the mean+standard deviation from at least three independent experiments performed in triplicate samples/condition. The *P*-values were determined using Student's t-test.

A

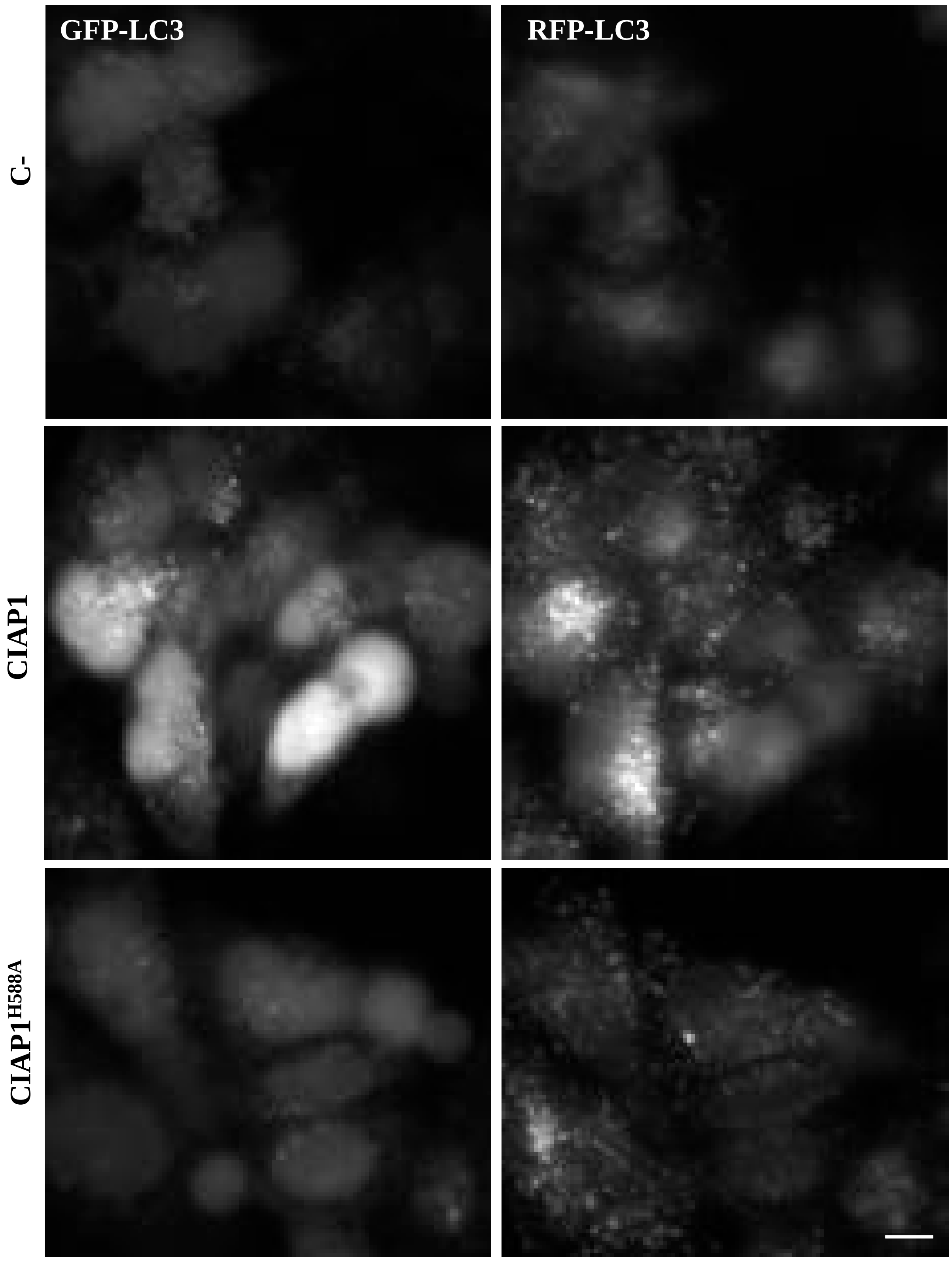

B

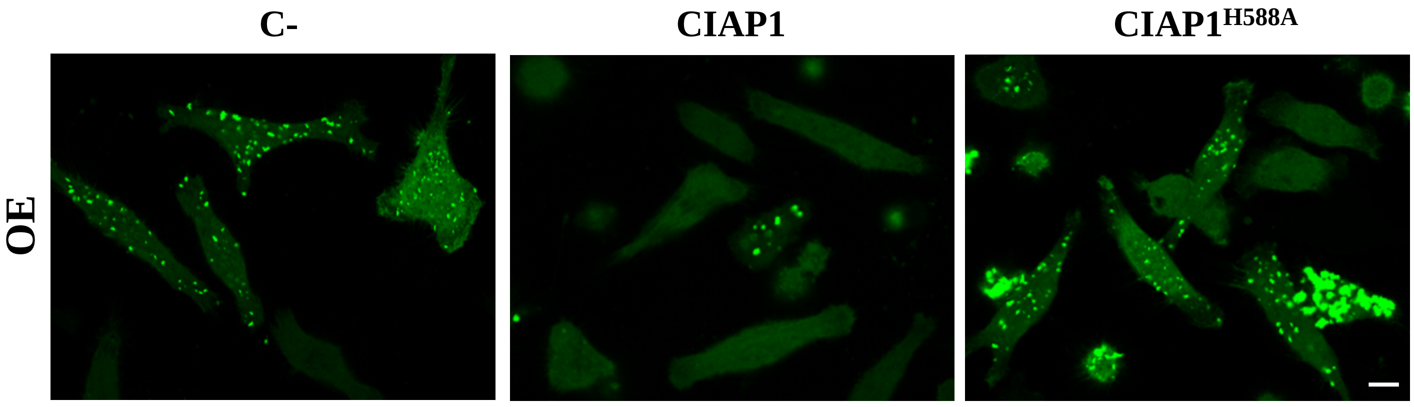

Fig. S9

Supplementary Fig. 9: Representative images for GFP-mRFP-LC3 dots and EGFP-HttQ74 aggregates under cIAP1 overexpression. (A) HeLa cells stably expressing mRFP-GFP-LC3 transfected with empty vector (C-) or CIAP 1 expression constructs **(C)** for 48 h were fixed and imaged using the Cellomics ArrayScan VTI HCS Reader (40x objective) and the Spot Detector V3 Cellomics BioApplication (Thermo Fisher Scientific). Note that these are not confocal images but were the images that were obtained from the automated microcope. **(B)** HeLa cells were cotransfected with the GFP-HttQ74 expression construct plus empty vector (C-) or CIAP expression constructs for 48 h. The cells were fixed and imaged using a confocal microscope Bar, 10 μm.

A

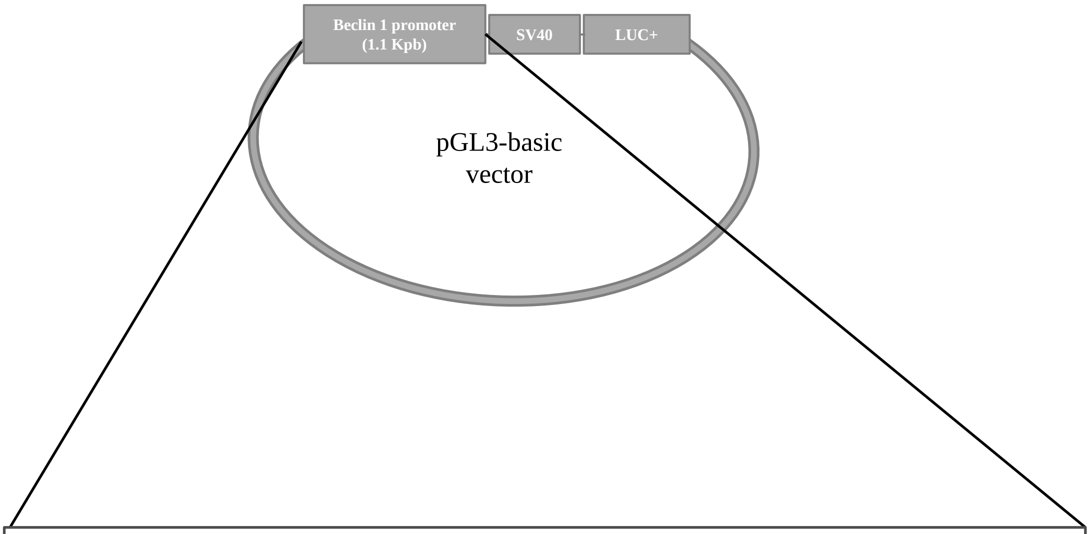

…CCTCCGCCTCCGGGGGTCAAGCGATTTTCCTACCCCAGCCTCCTGAGTAGCTGGGATTACAGGCGCACGC
CACCATGCCCAGCTAATTGTATTTTTTAGTAGAGACGGGATTTAACCATTTTGGCCAGGCTGGTCTCGAACTC
CTGACCTTGTGATCCGCCCGCCTCGGCCTCCCAAAGTGCTGGAATTACAAGCGTGAGCCACCATGCCCGGCC
TTTTGTTGTTGCTGTTGTTGTTCTGAGATGGAGCCTTGCCCTGTCGCCCAGGCTGGAGTGCAGTGGCCCGATC
TCGGCTCACTGCAACCTCCACCTCCCAGGTTCAAGCGATTCTCCTGCCTCAGCCTCCCGAGTAGCTGGGATT
AAGCTGGGATTATAGGCGTGCCCCACCACGCCCGGCTAGTTTTTGTATTTTTAGTAGAGACGGGGTTTCACT
GTGTTGGCCAGGCTGGTCTCGAACTCCTGACCTCACGTGATCCGCCCTCCTCGGCCTCCCCAAGTGCTGAGA
TTACAGGCGTGAGCCACCGCGCCCGCCGCCCCCTGAATTTAGAGAATAGCGGAGCCTCCCCATTCTCTGCGG
CCTTGGCTCCTACACTTCCCGTGGTAACCTTGTTCATCCGCTGAAGCCCGCTGCTTTTCCCAGCCCGGCCTCT
GGGGGCCGCTGCCGGGCCTGTGAGCCTGTGGACCAGGAGCTCCTGCTGCCGTCGTAGCGTCACGTCCGGTCT
CGGCGGAAGTTTTCCGGCGGCTACCGGGAAGTCGCTGAAGACAGAGCGATGGTAGTTCTGGAGGCCTCGCT
CCGGGGCCGACCCGAGGCCACAGTGCCTCCGCGGTAGACCGGACTTGGGTGACGGGCTCCGGGCTCCCGAG
GTGAAGAGCATCGGGGGCTGAGGTGGGACCTTAGAAGGGAGTCTGGGAACCCTCACGGCTCTTATTGGAGT
CCCTTCCCTGACCCTGGGCTCTAAACTGCCTTTGCTCAGGCTGTCCCGGAAGCAGGTCCTCCCCGTATCATAC
CATTCCTAGAGGGAACGGCGCAGGTTGGGACTTCCCTCCCTTTACCATCGTCACCAAGG…

B

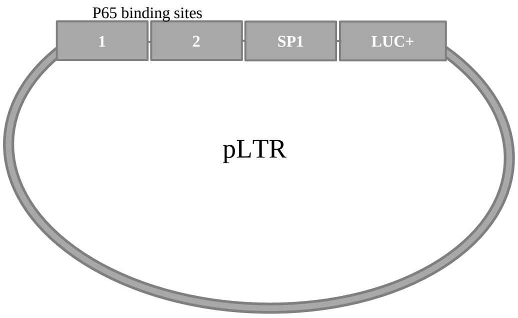

Fig. S10

Supplementary Fig. 10: XIAP induces Beclin 1 transcription *via* p65/NFκB activation. **(A)** Schematic diagram of pGL3-basic luciferase reporter vector containing an SV40 promoter to which a 1.1-kbp (CHET4) promoter region of the human beclin 1 gene encompassing p65 binding to this promoter was inserted (Copetti et al, 2009). **(B)** Schematic diagram of pLTRluc that contains two tandem p65 binding sites and three Sp1-binding sites (Pereira et al, 2000).
